# Essential roles of the cytokine oncostatin M in crosstalk between muscle fibers and immune cells in skeletal muscle after aerobic exercise

**DOI:** 10.1016/j.jbc.2022.102686

**Published:** 2022-11-09

**Authors:** Tadasuke Komori, Yoshihiro Morikawa

**Affiliations:** Department of Anatomy & Neurobiology, Wakayama Medical University, Wakayama, Japan

**Keywords:** exercise, skeletal muscle, cytokine, macrophage, neutrophil, chemokine, CCL, CC chemokine ligand, CXCL1, CXC chemokine ligand 1, CX3CL1, CX3C chemokine ligand 1, DMEM, Dulbecco's modified Eagle's medium, IgG, immunoglobulin G, IL, interleukin, iNOS, inducible nitric oxide synthase, NIH, National Institutes of Health, OSM, oncostatin M, OSMRβ, OSM receptor β subunit, PE, phycoerythrin, PPARδ, peroxisome proliferator–activated receptor δ, RT, room temperature, STAT3, signal transducer and activator of transcription 3, TNF-α, tumor necrosis factor alpha

## Abstract

Crosstalk between muscle fibers and immune cells is well known in the processes of muscle repair after exercise, especially resistance exercise. In aerobic exercise, however, this crosstalk is not fully understood. In the present study, we found that macrophages, especially anti-inflammatory (M2) macrophages, and neutrophils accumulated in skeletal muscles of mice 24 h after a single bout of an aerobic exercise. The expression of oncostatin M (OSM), a member of the interleukin 6 family of cytokines, was also increased in muscle fibers immediately after the exercise. In addition, we determined that deficiency of OSM in mice inhibited the exercise-induced accumulation of M2 macrophages and neutrophils, whereas intramuscular injection of OSM increased these immune cells in skeletal muscles. Furthermore, the chemokines related to the recruitment of macrophages and neutrophils were induced in skeletal muscles after aerobic exercise, which were attenuated in OSM-deficient mice. Among them, CC chemokine ligand 2, CC chemokine ligand 7, and CXC chemokine ligand 1 were induced by OSM in skeletal muscles. Next, we analyzed the direct effects of OSM on the skeletal muscle macrophages, because the OSM receptor β subunit was expressed predominantly in macrophages in the skeletal muscle. OSM directly induced the expression of these chemokines and anti-inflammatory markers in the skeletal muscle macrophages. From these findings, we conclude that OSM is essential for aerobic exercise–induced accumulation of M2 macrophages and neutrophils in the skeletal muscle partly through the regulation of chemokine expression in macrophages.

Physical inactivity in a sedentary lifestyle is a leading risk factor for various noncommunicable diseases, including obesity, type 2 diabetes, hypertension, and coronary heart disease (1). Physical inactivity is well known to have a global prevalence, and there have been various efforts in recent years to counteract this problem (2). In addition to pharmacological interventions in the treatment and prevention of noncommunicable diseases, regular exercise is recommended for preventing, managing, and treating physical inactivity and the related chronic conditions (1). Exercise is a specific form of physical activity, which is carried out for a set purpose, such as health benefits, in a structured, planned, and continuous (repetitive) manner (3, 4). Benefits of exercise have long been well known, but such exercise interventions require the understanding of precise mechanisms by which exercise and physical activity prevent and/or improve these conditions.

Exercises are generally grouped into types depending on the modality (aerobic *versus* resistance) and the intensity, duration, and frequency of training session. Each type of exercise has unique health benefits through differing skeletal muscle adaption to it. Aerobic exercise, such as brisk walking, jogging, and swimming, is most commonly used in the treatment and prevention of noncommunicable diseases (4, 5), whereas resistance exercise requires muscle contraction against external resistance in order to improve the strength and mass of the muscles (5). Skeletal muscles release a diverse range of biological active peptides and proteins, so-called myokines (6). Regardless of the type of exercise, muscle contraction during exercise is a major stimulus of the expression and secretion of myokines (6), and therefore, numerous myokines, such as interleukin 6 (IL-6) family members, IL-6 (7), and leukemia inhibitory factor (8), are well known to be produced in skeletal muscles after a bout of acute exercise. Myokines are secreted into the blood circulation to confer the beneficial health effects of exercise to other organs, including to adipose tissue and the liver (6). In addition to their extramuscular effects, some myokines, including IL-6, have regulatory function of glucose and lipid metabolism within the skeletal muscle itself (9).

Oncostatin M (OSM), a member of IL-6 family of cytokines (10), activates the heterodimeric membrane receptor comprising the OSM receptor β subunit (OSMRβ) and the common receptor subunit, gp130 (11). OSM has a variety of biological effects, such as hematopoiesis (12), development of the liver (13), and modulation of inflammation (14). We and others (15, 16) have demonstrated that treatment with OSM improves some noncommunicable diseases, including obesity, type 2 diabetes, and coronary heart disease. We have also reported that OSM produced by adipocytes has the ability to improve insulin resistance with obesity through the phenotypic change of macrophages from proinflammatory (M1) to anti-inflammatory (M2) type in adipose tissue (17). In addition, Hojman *et al.* (18) reported that a single bout of swimming, a type of aerobic exercise, induces the expression of OSM in skeletal muscle. However, the roles of OSM produced in skeletal muscle during aerobic exercise have not been sufficiently elucidated.

Obesity-related metabolic disorders, including type 2 diabetes and hepatic steatosis, are associated with low-grade chronic systemic inflammation (19). In the obese state, various inflammatory cells are recruited in the white adipose tissue (20) and skeletal muscle (21). Recruited inflammatory cells, especially M1 macrophages, release proinflammatory cytokines, such as tumor necrosis factor alpha (TNF-α) and IL-1β, resulting in insulin resistance (22, 23, 24). On the other hand, M2 macrophages are well known to play an important role in these tissues on the beneficial effects to obesity-related metabolic disorders, including enhancement of insulin sensitivity (24). Exercise induces M1-to-M2 phenotype switch of macrophages in multiple organs, including in skeletal muscle, liver, and adipose tissue (25). Accumulation of M2 macrophages enhances insulin sensitivity in skeletal muscle after a single bout of aerobic exercise (26), but the mechanism of M2 macrophage accumulation after aerobic exercise is largely unknown.

In the present study, we investigated cellular and molecular events in the skeletal muscle after a single bout of aerobic exercise and the roles of OSM in the crosstalk between muscle fibers and immune cells.

## Results

### Accumulation of macrophages and neutrophils in skeletal muscle after a single bout of aerobic exercise

**Figure 1:**
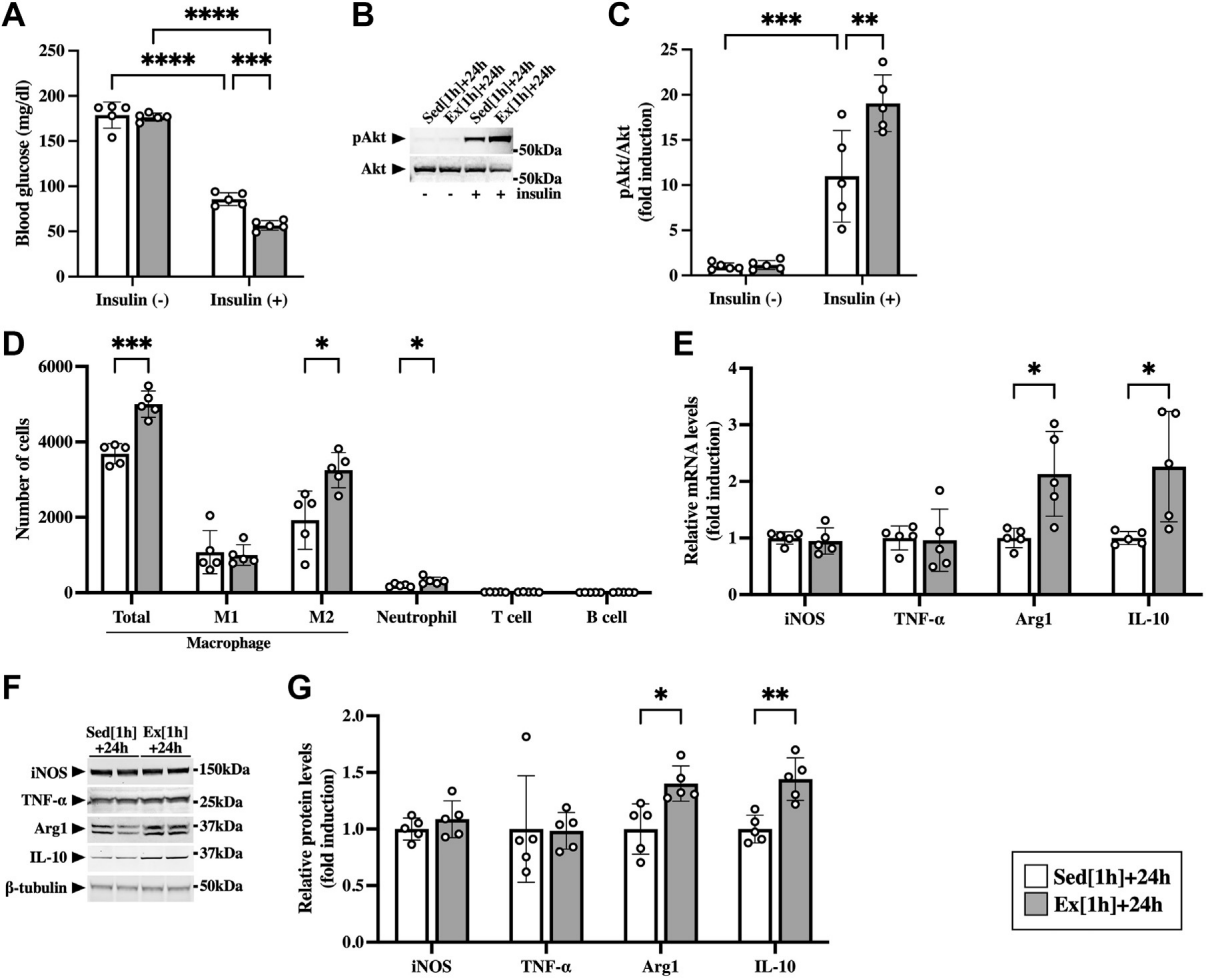
**Accumulation of immune cells in the skeletal muscle after the aerobic exercise.** Male C57BL/6J mice were subjected to a single bout of treadmill running exercise at a speed of 15 m/min for 1 h. Mice in the sedentary group were used as controls. *A*–*C*, at 24 h after the exercise, mice were intraperitoneally injected with insulin (1 milliunit/g of body weight). *A*, insulin-induced decreases in blood glucose in sedentary mice (Sed [1 h] + 24 h) and mice at 24 h after the exercise (Ex [1 h] + 24 h). *B*, Western blot analysis of phospho-Akt (pAkt) in the skeletal muscle. The apparent molecular weights are indicated on the *right*. *C*, quantitative analysis of pAkt. The band intensities of pAkt were normalized to total Akt and represented as the fold induction relative to the intensities of sedentary mice with no insulin injection in the bar graph. *D*, flow cytometric analysis of immune cells in the skeletal muscle at 24 h after the exercise. *E*, gene expression of M1 (iNOS and TNF-α) and M2 markers (arginase-1 [Arg1] and IL-10) in the skeletal muscle at 24 h after the exercise. *F*, Western blot analysis of M1 (iNOS and TNF-α) and M2 markers (arginase-1 [Arg1] and IL-10) in the skeletal muscle at 24 h after the exercise. The apparent molecular weights are indicated on the *right*. *G*, quantitative analysis of the protein expression of iNOS, TNF-α, Arg1, and IL-10. The band intensities of iNOS, TNF-α, Arg1, and IL-10 were normalized to β-tubulin and represented as the fold induction relative to the intensities of sedentary mice in the bar graph. Data are expressed as mean ± SD; n = 5 per group. ∗*p* < 0.05, ∗∗*p* < 0.01, ∗∗∗*p* < 0.001, and ∗∗∗∗*p* < 0.0001. Two-way ANOVA followed by Tukey’s post hoc test (*A* and *C*); Student’s *t* test (*D*, *E*, and *G*). IL, interleukin; iNOS, inducible nitric oxide synthase; TNF-α, tumor necrosis factor alpha.

First, we tested whether a single bout of aerobic exercise enhances muscle insulin sensitivity 24 h after the exercise. No significant differences in blood glucose levels were found between sedentary mice and mice after the exercise (Fig. 1*A*). Insulin lowered blood glucose levels (Fig. 1*A*) and induced phosphorylation of Akt in the skeletal muscle (Fig. 1, *B* and *C*). In addition, such effects of insulin were significantly enhanced by the exercise (Fig. 1, *A*–*C*), suggesting that a single bout of aerobic exercise enhanced insulin sensitivity in the skeletal muscle.

In this context, we examined the effects of a single bout of aerobic exercise on accumulation of immune cells in the skeletal muscle by using flow cytometry. Both total macrophages (CD45^+^/CD11b^+^/F4/80^+^) and neutrophils (CD45^+^/CD11b^+^/Gr-1^high^) were increased in the skeletal muscle after exercise (Fig. 1*D*). In total macrophages, M2 macrophages (CD45^+^/CD11b^+^/F4/80^+^/CD206^+^) were exclusively increased in the skeletal muscle (Fig. 1*D*). The exercise had no significant effects on the numbers of M1 macrophages (CD45^+^/CD11b^+^/F4/80^+^/CD11c^+^), T cells (CD45^+^/CD3e^+^), and B cells (CD45^+^/B220^+^) in the skeletal muscle (Fig. 1*D*). To investigate the distribution of accumulated immune cells in the skeletal muscle (gastrocnemius, plantaris, and soleus muscles), immunohistological analysis was performed 24 h after the exercise. Consistent with the data obtained from flow cytometry, both total (F4/80-positive cells) and M2 macrophages (CD206-positive cells) were increased in the skeletal muscle after the exercise (Fig. S1). Similar patterns of immune cell alteration were observed between the gastrocnemius, plantaris, and soleus muscles (Fig. S1). In addition, both mRNA and protein expression of markers of M2 macrophage, arginase-1 and IL-10, were increased in the skeletal muscle 24 h after the exercise (Fig. 1, *E*–*G*). There were no significant differences, however, between the sedentary and exercise groups in the expression of markers of M1 macrophages, inducible nitric oxide synthase (iNOS) or TNF-α, at both mRNA and protein levels (Fig. 1, *E*–*G*).

### Expression of OSM in the skeletal muscle after the aerobic exercise

**Figure 2:**
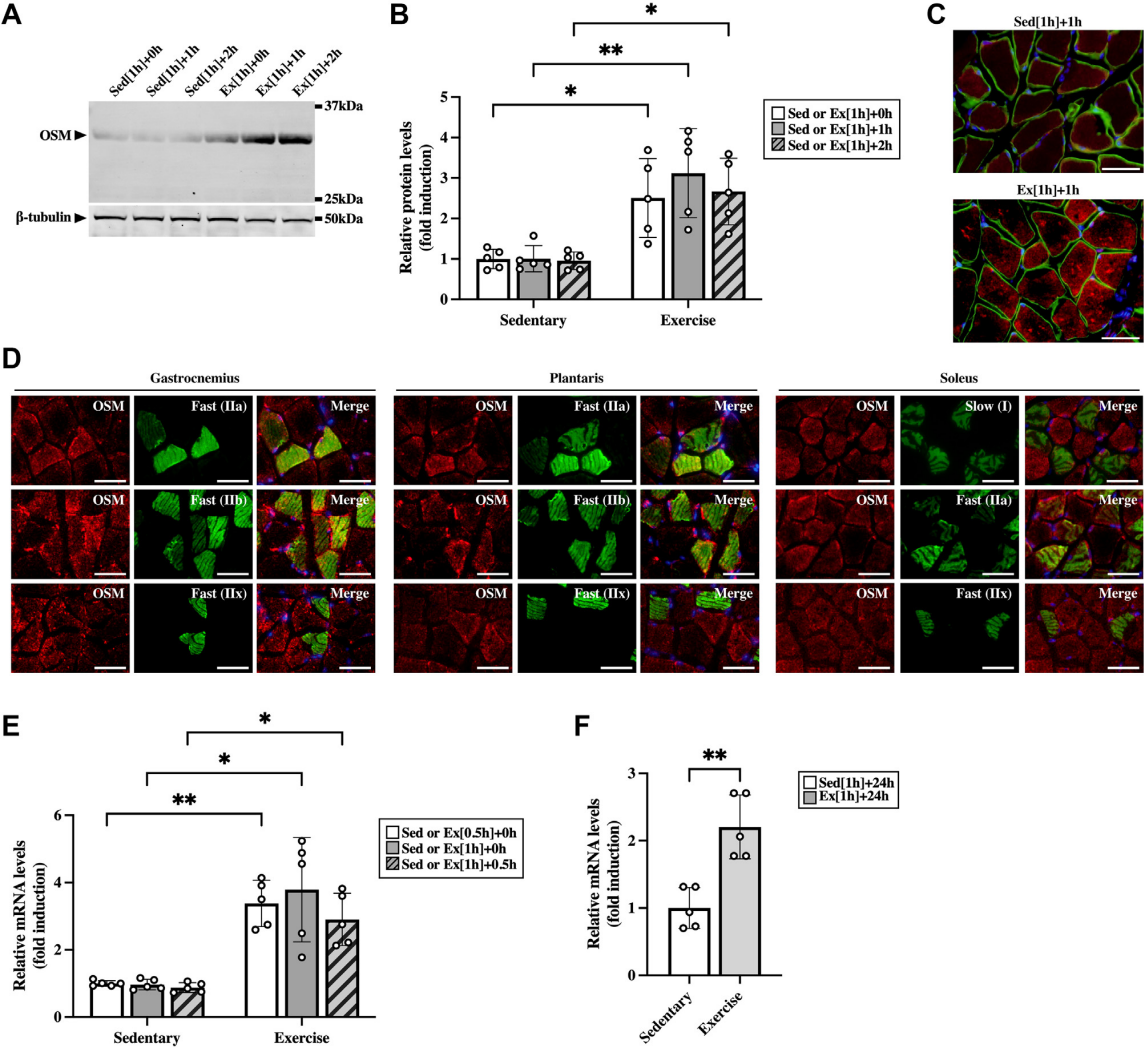
**The effects of aerobic exercise on the expression of OSM in the skeletal muscle.** Male C57BL/6J mice were subjected to a single bout of treadmill running exercise at a speed of 15 m/min for 0.5 or 1 h. Mice in the sedentary group were used as controls. *A*, Western blot analysis of OSM in the skeletal muscle of mice immediately (Ex [1 h] + 0 h), 1 h (Ex [1 h] + 1 h), and 2 h (Ex [1 h] + 2 h) after the exercise and each sedentary control (Sed [1 h] + 0 h, Sed [1 h] + 1 h, and Sed [1 h] + 2 h). The apparent molecular weights are indicated on the *right*. *B*, quantitative analysis of the protein expression of OSM. The band intensities of OSM were normalized to β-tubulin and represented as the fold induction relative to the intensities on each sedentary group. *C*, localization of OSM protein in the skeletal muscle at 1 h after the exercise. Sections were stained with an antibody against OSM (*red*) and costained with an antibody against laminin to identify the muscle fibers (*green*). The scale bars represent 50 μm. *D*, muscle fiber type distribution of exercise-induced OSM in the skeletal muscle. Sections were stained with an antibody against OSM (*red*) and costained with antibodies against myosin heavy chain type I, IIa, IIb, or IIx to identify the muscle fiber types (*green*). *C* and *D*, the sections were counterstained with DAPI (*blue*). The scale bars represent 50 μm. *E* and *F*, gene expression of OSM in the skeletal muscle of mice at 0.5 h from the start of the exercise (Ex [0.5 h] + 0 h), and immediately (Ex [1 h] + 0 h), 0.5 h (Ex [1 h] + 0.5 h), and 24 h (Ex [1 h] + 24 h) after 1 h of exercise. Data are expressed as mean ± SD; n = 5 per group. ∗*p* < 0.05 and ∗∗*p* < 0.01. Two-way ANOVA followed by Tukey’s post hoc test (*B* and *E*); Student’s *t* test (*F*). DAPI, 4′,6-diamidino-2-phenylindole; OSM, oncostatin M.

To assess the roles of OSM in exercise-induced alteration of immune cells, we first examined the effects of the aerobic exercise on the expression of OSM in the skeletal muscle. The protein expression of OSM was induced in the skeletal muscle immediately after exercise and remained at a high level until 2 h after the exercise (Fig. 2, *A* and *B*). To eliminate the possibility that OSM was upregulated by the electrical shock used to encourage running on the treadmill, we examined the expression of OSM in the skeletal muscle after swimming exercise and treadmill running exercise with gentle encouragement by using a tongue depressor. In both exercise protocols, OSM was increased in the skeletal muscle immediately after the exercise (Fig. S2). To identify OSM-expressing cells in the skeletal muscle after exercise, immunohistological analysis was performed 1 h after the exercise. Intense expression of OSM protein was observed in the muscle fibers after the exercise (Fig. 2*C*). In addition, OSM was widely distributed in both slow-twitch fibers (type I) and fast-twitch fibers (types IIa, IIb, and IIx) after the exercise (Fig. 2*D*).

To investigate whether the exercise induced protein expression of OSM because of the increase of gene expression of OSM, we examined the gene expression of OSM in the skeletal muscle during and after exercise. The mRNA expression of OSM began to increase at 0.5 h from the start of the exercise and remained at high level immediately after 1 h of exercise and at 0.5 h after the exercise (Fig. 2*E*). In addition, OSM expression was still high in the skeletal muscle at 24 h after the exercise (Fig. 2*F*).

### Alteration of macrophages and neutrophils in skeletal muscle of OSM-deficient mice after aerobic exercise

**Figure 3:**
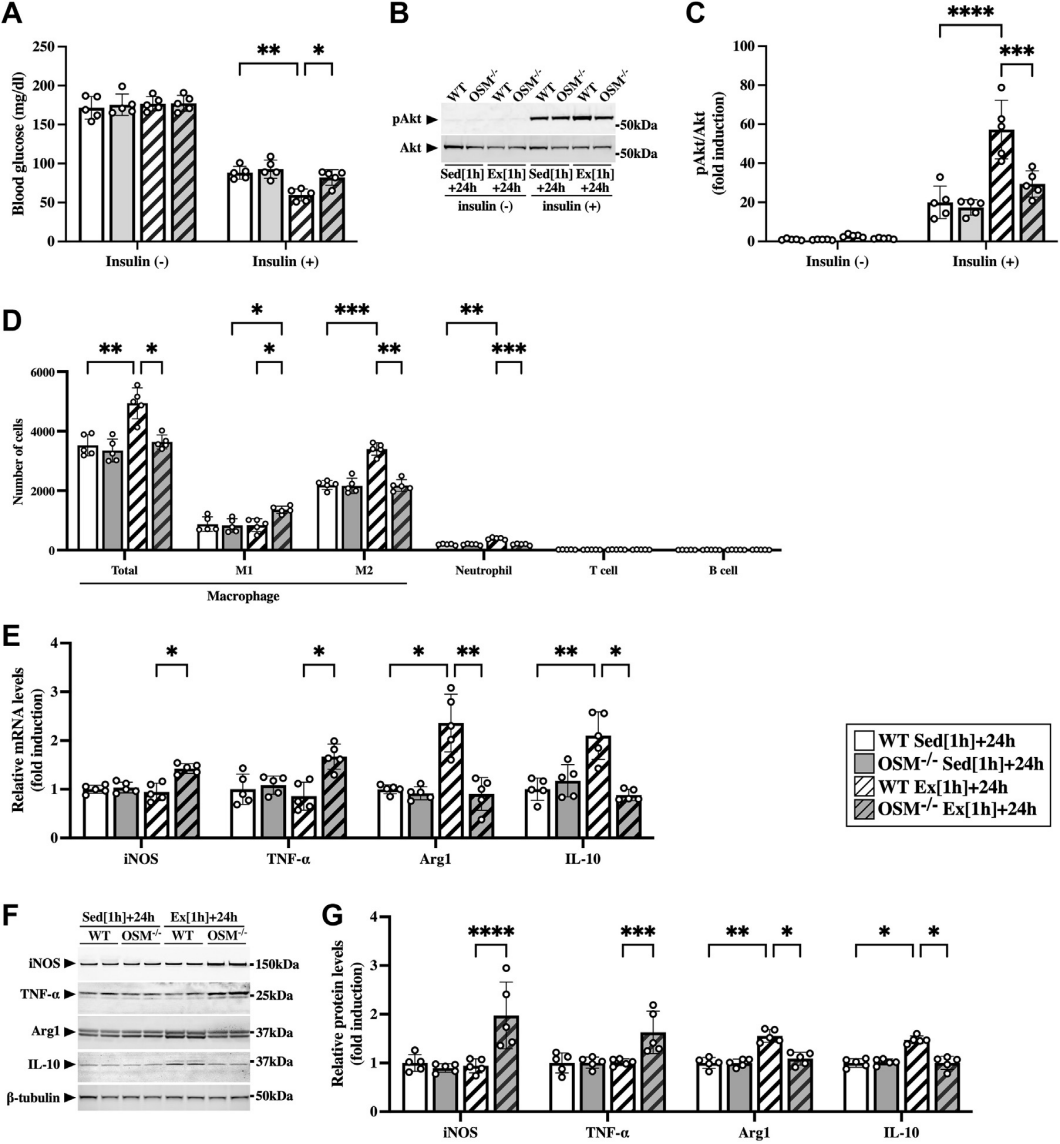
**Effects of OSM deficiency on the accumulation of macrophages and neutrophils in the skeletal muscle after the aerobic exercise.** Male WT and OSM^−/−^ mice were subjected to a single bout of treadmill running exercise at a speed of 15 m/min for 1 h. Mice in the sedentary group were used as controls. *A*–*C*, at 24 h after the exercise, mice were intraperitoneally injected with insulin (1 milliunits/g of body weight). *A*, insulin-induced decreases in blood glucose in sedentary WT mice (WT Sed [1 h] + 24 h), sedentary OSM^−/−^ mice (OSM^−/−^ Sed [1 h] + 24 h), WT mice at 24 h after the exercise (WT Ex [1 h] + 24 h), and OSM^−/−^ mice at 24 h after the exercise (OSM^−/−^ Ex [1 h] + 24 h). *B*, Western blot analysis of pAkt in the skeletal muscle. The apparent molecular weights are indicated on the *right*. *C*, quantitative analysis of pAkt. The band intensities of pAkt were normalized to total Akt and are represented as the fold induction relative to the intensities of WT mice in the bar graph. *D*, flow cytometric analysis of immune cells in the skeletal muscle of WT and OSM^−/−^ mice at 24 h after the exercise. *E*, gene expression of M1 (iNOS and TNF-α) and M2 markers (arginase-1 [Arg1] and IL-10) in the skeletal muscle of WT and OSM^−/−^ mice at 24 h after the exercise. *F*, Western blot analysis of M1 (iNOS and TNF-α) and M2 markers (arginase-1 [Arg1] and IL-10) in the skeletal muscle of WT and OSM^−/−^ mice at 24 h after the exercise. The apparent molecular weights are indicated on the *right*. *G*, quantitative analysis of the protein expression of iNOS, TNF-α, Arg1, and IL-10. The band intensities of iNOS, TNF-α, Arg1, and IL-10 were normalized to β-tubulin and are represented as the fold induction relative to the intensities of WT mice in the bar graph. Data are expressed as mean ± SD; n = 5 per group. ∗*p* < 0.05, ∗∗*p* < 0.01, ∗∗∗*p* < 0.001, and ∗∗∗∗*p* < 0.0001. Two-way ANOVA followed by Tukey’s post hoc test. IL, interleukin; iNOS, inducible nitric oxide synthase; OSM, oncostatin M; TNF-α, tumor necrosis factor alpha.

Next, we investigated the roles of OSM in exercise-induced metabolic changes and immune cell alterations by using OSM-deficient (OSM^−/−^) mice. Under sedentary condition, there were no significant differences between WT and OSM^−/−^ mice in the blood glucose levels (Fig. 3*A*), Akt phosphorylation (Fig. 3, *B* and *C*), the number of immune cells (Fig. 3*D*), or the expression of M1 and M2 markers (Fig. 3, *E*–*G*). After the exercise, there were no significant differences between WT and OSM^−/−^ mice in blood glucose levels before insulin injection (Fig. 3*A*). Although insulin decreased blood glucose levels in both WT and OSM^−/−^ mice after the exercise, the reduction in blood glucose with insulin was lower in OSM^−/−^ mice than in WT mice (Fig. 3*A*). In addition, insulin-stimulated phosphorylation of Akt was significantly attenuated in the skeletal muscle of OSM^−/−^ mice compared with that in WT mice (Fig. 3, *B* and *C*). Flow cytometry revealed that both total macrophages and neutrophils were decreased in the skeletal muscle of OSM^−/−^ mice compared with those in WT mice after exercise (Fig. 3*D*). In total macrophages, M2 macrophages were decreased, whereas M1 macrophages were increased in the skeletal muscle of OSM^−/−^ mice after the exercise (Fig. 3*D*). There were no significant changes in the numbers of T cells and B cells in the skeletal muscle after the exercise (Fig. 3*D*). The alteration patterns of the number of immune cells observed in flow cytometry were confirmed in all part of calf muscles (gastrocnemius, plantaris, and soleus) after the exercise (Fig. S3). Both mRNA and protein expression of M2 markers (arginase-1 and IL-10) were decreased, and M1 markers (iNOS and TNF-α) were increased in the skeletal muscle of OSM^−/−^ mice at 24 h after the exercise (Fig. 3, *E*–*G*).

### Effects of OSM on alteration of macrophages and neutrophils in skeletal muscle

**Figure 4:**
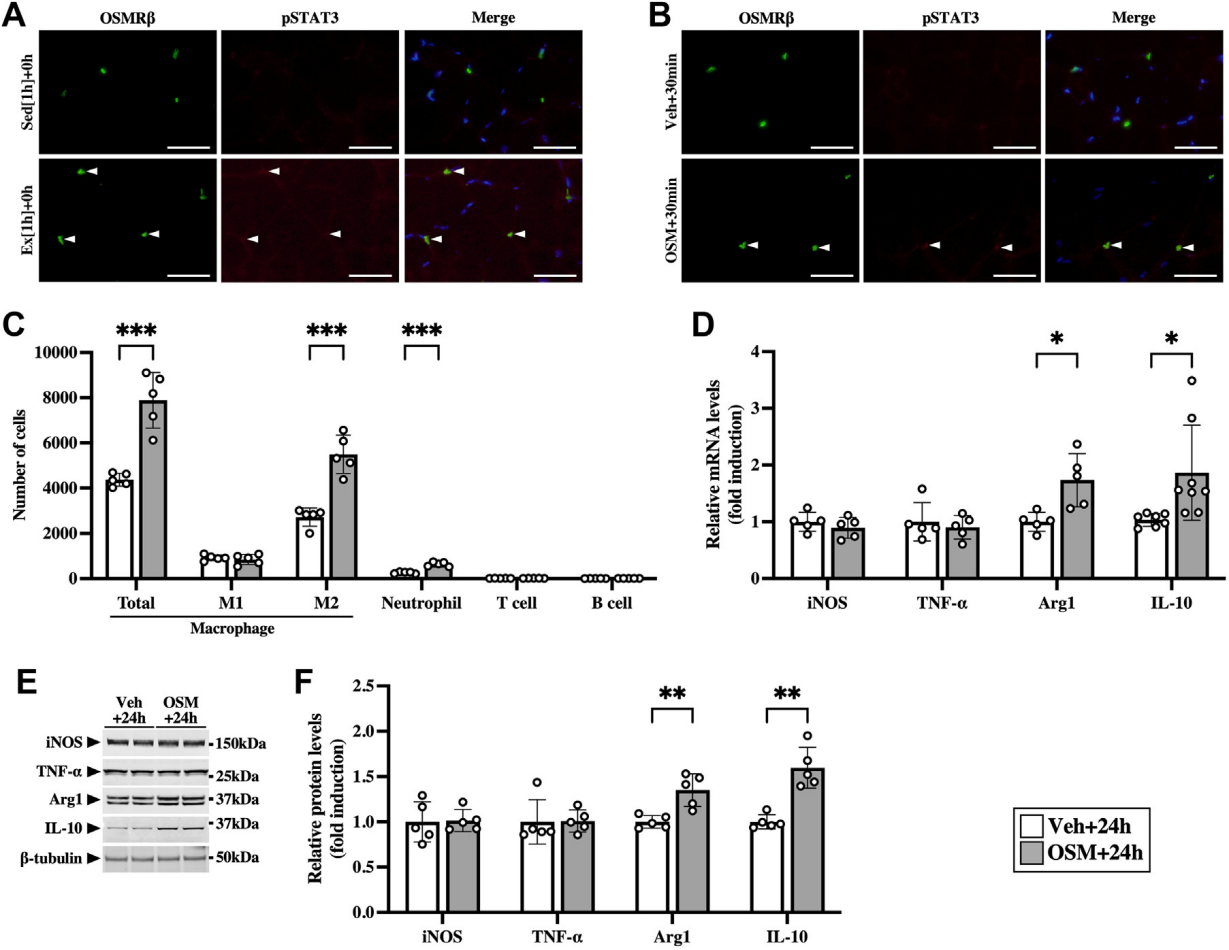
**Effects of OSM on the accumulation of macrophages and neutrophils in the skeletal muscle.***A*, exercise-induced phosphorylation of STAT3 (pSTAT3) in OSMRβ-positive cells in the skeletal muscle immediately after the exercise. *Arrowheads* indicate OSMRβ (*left panels*) and pSTAT3 (*middle panels*) double-positive cells. The merged images are shown in the *right panels*. The sections were counterstained with DAPI (*blue*). The scale bars represent 50 μm. *B*–*F*, male C57BL/6J mice were injected intramuscularly with vehicle (Veh) or recombinant mouse OSM. *B*, OSM-induced pSTAT3 in OSMRβ-positive cells in the skeletal muscle at 30 min after the injection. *Arrowheads* indicate OSMRβ (*left panels*) and pSTAT3 (*middle panels*) double-positive cells. The merged images are shown in the *right panels*. The sections were counterstained with DAPI (*blue*). The scale bars represent 50 μm. *C*, flow cytometric analysis of immune cells in the skeletal muscle at 24 h after the injection of OSM (OSM + 24 h) and its controls (Veh + 24 h). *D*, gene expression of M1 (iNOS and TNF-α) and M2 markers (arginase-1 [Arg1] and IL-10) in the skeletal muscle at 24 h after the injection. *E*, Western blot analysis of M1 (iNOS and TNF-α) and M2 markers (arginase-1 [Arg1] and IL-10) in the skeletal muscle at 24 h after the injection. The apparent molecular weights are indicated on the *right*. *F*, quantitative analysis of the protein expression of iNOS, TNF-α, Arg1, and IL-10. The band intensities of iNOS, TNF-α, Arg1, and IL-10 were normalized to β-tubulin and are represented as the fold induction relative to the intensities of Veh group in the bar graph. Data are expressed as mean ± SD; n = 5 per group. ∗*p* < 0.05, ∗∗*p* < 0.01, and ∗∗∗*p* < 0.001. Student’s *t* test. DAPI, 4′,6-diamidino-2-phenylindole; IL, interleukin; iNOS, inducible nitric oxide synthase; OSM, oncostatin M; OSMRβ, OSM receptor β subunit; TNF-α, tumor necrosis factor alpha.

Double immunofluorescence staining of OSMRβ with phosphorylated signal transducer and activator of transcription 3 (STAT3) was performed after the exercise because STAT3 is phosphorylated by OSM through OSMRβ–gp130 receptor complex. Exercise induced phosphorylation of STAT3 in OSMRβ-positive cells (Fig. 4*A*), suggesting the possibility that OSMRβ-positive cells were activated by OSM.

To investigate whether exercise-induced immune cell alterations were reproduced by OSM alone in the skeletal muscle without the exercise, OSM was injected intramuscularly. Phosphorylated STAT3 was detected in OSMRβ-positive cells at 30 min after the injection of OSM (Fig. 4*B*). At 24 h after the injection, total macrophages, M2 macrophages, and neutrophils were increased in the skeletal muscle (Fig. 4*C*). OSM had no significant effects on the numbers of M1 macrophages, T cells, and B cells in the skeletal muscle (Fig. 4*C*). Similar alteration patterns of the number of immune cells were observed in all part of calf muscles (gastrocnemius, plantaris, and soleus) after the injection of OSM (Fig. S4). The expression of M2 markers (arginase-1 and IL-10) was increased by OSM, whereas M1 markers (iNOS and TNF-α) were not significantly changed at the mRNA (Fig. 4*D*) and protein levels (Fig. 4, *E* and *F*).

### Effects of OSM on chemokine production in skeletal muscle

**Figure 5:**
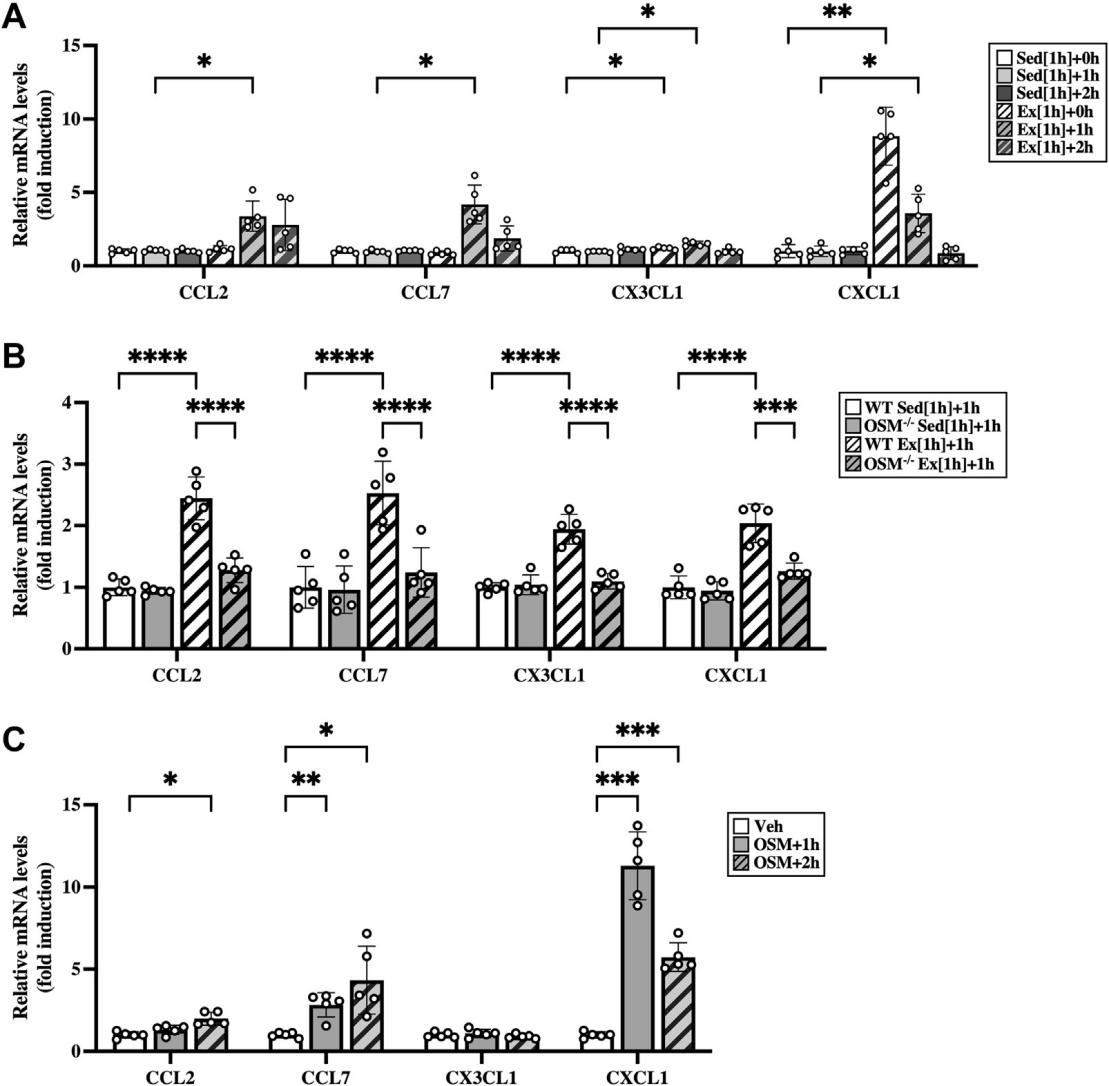
**Effects of OSM on the mRNA expression of exercise-induced chemokines in the skeletal muscle.***A*, expression of chemokines in the skeletal muscle after the aerobic exercise. Male C57BL/6J mice were subjected to a single bout of treadmill running exercise at a speed of 15 m/min for 1 h. Mice in the sedentary group were used as controls. *B*, the expression of chemokines in the skeletal muscle of WT and OSM^−/−^ mice at 1 h after the exercise. Male WT and OSM^−/−^ mice were subjected to a single bout of treadmill running exercise at a speed of 15 m/min for 1 h. *C*, effects of OSM on the expression of chemokines in the skeletal muscle of mice at 1 or 2 h after the intramuscular injection of vehicle or recombinant mouse OSM (12.5 ng/g body weight). Data are expressed as mean ± SD; n = 5 per group. ∗*p* < 0.05, ∗∗*p* < 0.01, ∗∗∗*p* < 0.001, and ∗∗∗∗*p* < 0.0001. Two-way ANOVA followed by Tukey’s post hoc test. CCL, CC chemokine ligand; CXCL1, CXC chemokine ligand 1; OSM, oncostatin M.

To better understand which chemokines contribute to the accumulation of M2 macrophages and neutrophils in the skeletal muscle after aerobic exercise, we investigated the mRNA expression of CC chemokine ligand (CCL) 2, CCL7, CX3C chemokine ligand 1 (CX3CL1), and CXC chemokine ligand (CXCL1) in the skeletal muscle after the exercise. The chemokines associated with monocyte recruitment, CCL2, CCL7, and CX3CL1, were induced by exercise in the skeletal muscle, and their expression levels were highest at 1 h after exercise (Fig. 5*A*). CXCL1, a chemokine for induction of neutrophils, increased immediately after exercise in the skeletal muscle (Fig. 5*A*). These chemokines were not increased in the adipose tissue (Fig. S5*A*) or the liver (Fig. S5*B*) at the time points after the exercise. The expression of CCL2, CCL7, CX3CL1, and CXCL1, which were induced by the exercise in WT mice, were attenuated in the skeletal muscle of OSM^−/−^ mice at 1 h after exercise (Fig. 5*B*). In addition, intramuscular injection with OSM induced the expression of CCL2, CCL7, and CXCL1 in the skeletal muscle (Fig. 5*C*). The expression of CX3CL1, however, was not induced by OSM in the skeletal muscle (Fig. 5*C*).

**Figure 6:**
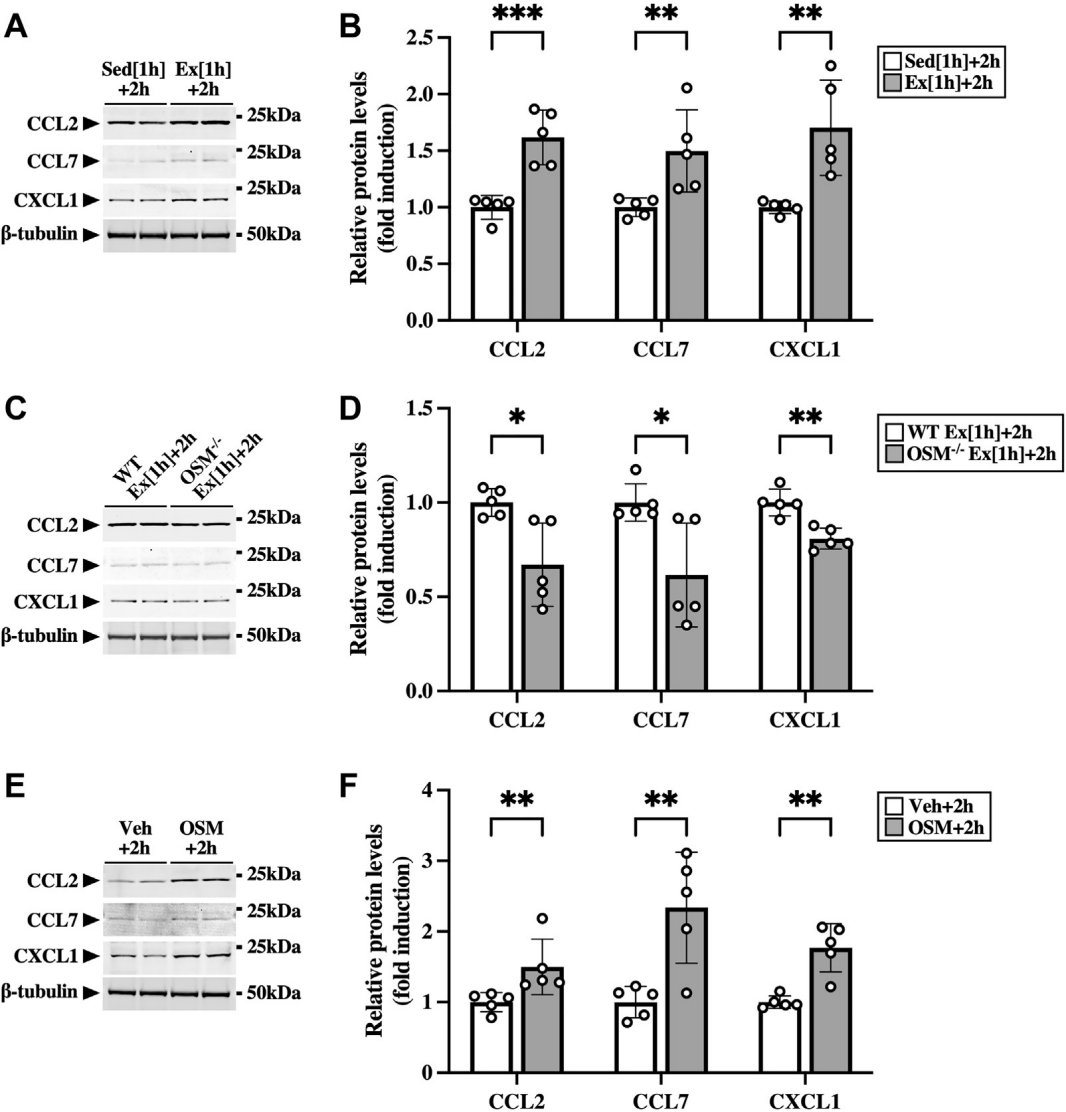
**Effects of OSM on the protein expression of exercise-induced chemokines in the skeletal muscle.***A*, Western blot analysis of CCL2, CCL7, and CXCL1 in the skeletal muscle of sedentary mice (Sed [1 h] + 2 h) and mice at 2 h after the exercise (Ex [1 h] + 2 h). The apparent molecular weights are indicated on the *right*. *B*, quantitative analysis of the protein expression of CCL2, CCL7, and CXCL1. The band intensities of CCL2, CCL7, and CXCL1 were normalized to β-tubulin and are represented as the fold induction relative to the intensities of sedentary mice in the bar graph. *C*, Western blot analysis of CCL2, CCL7, and CXCL1 in the skeletal muscle of WT and OSM^−/−^ mice at 2 h after the exercise. The apparent molecular weights are indicated on the *right*. *D*, quantitative analysis of the protein expression of CCL2, CCL7, and CXCL1. The band intensities of CCL2, CCL7, and CXCL1 were normalized to β-tubulin and are represented as the fold induction relative to the intensities of WT mice in the bar graph. *E*, Western blot analysis of CCL2, CCL7, and CXCL1 in the skeletal muscle of mice at 2 h after the injection of vehicle (Veh) or OSM. *F*, quantitative analysis of the protein expression of CCL2, CCL7, and CXCL1. The band intensities of CCL2, CCL7, and CXCL1 were normalized to β-tubulin and are represented as the fold induction relative to the intensities of Veh group in the bar graph. Data are expressed as mean ± SD; n = 5 per group. ∗*p* < 0.05, ∗∗*p* < 0.01, and ∗∗∗*p* < 0.001. Student’s *t* test. CCL, CC chemokine ligand; CXCL1, CXC chemokine ligand 1; OSM, oncostatin M.

To confirm the protein expression of CCL2, CCL7, and CXCL1, we performed Western blot analysis using the skeletal muscle at 2 h after the exercise. The protein expression of CCL2, CCL7, and CXCL1 was increased by the exercise (Fig. 6, *A* and *B*), which was attenuated in OSM^−/−^ mice (Fig. 6, *C* and *D*). In addition, intramuscular injection with OSM also induced the protein expression of these chemokines in the skeletal muscle (Fig. 6, *E* and *F*).

### Direct effects of OSM on skeletal muscle macrophages

**Figure 7:**
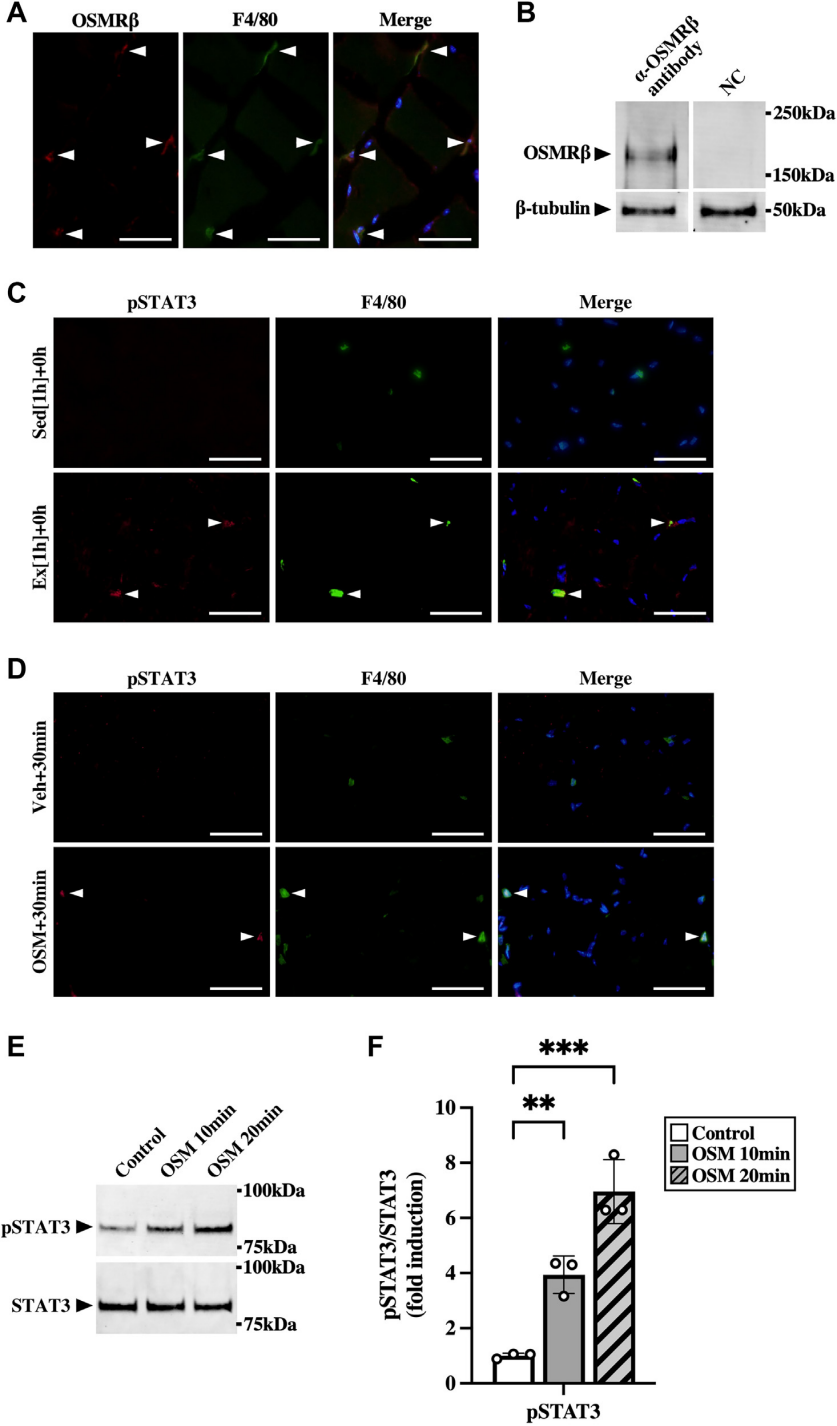
**OSM acts on macrophages in the skeletal muscle.***A*, localization of OSMRβ in the skeletal muscle of C57BL/6J mice. Sections were stained with antibodies against OSMRβ (*red*) and F4/80 (*green*). The sections were counterstained with DAPI (*blue*). Merged image is shown in the *right panel*. The scale bars represent 50 μm. *B*, Western blot analysis of OSMRβ in the isolated skeletal muscle macrophages of C57BL/6J mice. The apparent molecular weights are indicated on the *right*. In negative control (NC), the blotted membranes were incubated with goat immunoglobulin G (IgG) purified from normal goat serum instead of anti-OSMRβ antibody. *C*, localization of exercise-induced pSTAT3 in the skeletal muscle immediately after the exercise. Sections were stained with antibodies against pSTAT3 (*red*) and F4/80 (*green*). *Arrowheads* indicate pSTAT3 (*left panels*) and F4/80 (*middle panels*) double-positive cells. The scale bars represent 50 μm. *D*, effects of OSM on phosphorylation of STAT3 (pSTAT3) in skeletal muscle macrophages. C57BL/6J mice were given an injection with vehicle (*upper panels*) or recombinant mouse OSM (*lower panels*) intramuscularly at 30 min before the experiments. *Arrowheads* indicate pSTAT3 (*left panels*) and F4/80 (*middle panels*) double-positive cells. The merged images are shown in the *right panels*. The sections were counterstained with DAPI (*blue*). The scale bars represent 50 μm. *E*, Western blot analysis of pSTAT3 in isolated skeletal muscle macrophages by stimulation with OSM (50 ng/ml) for 10 or 20 min. The apparent molecular weights are indicated on the *right*. *F*, quantitative analysis of the pSTAT3. The band intensities of pSTAT3 were normalized to STAT3 and are represented as the fold induction relative to the intensities of the controls (*white bar*) in the bar graph. Data are expressed as mean ± SD; n = 3 per group. ∗∗*p* < 0.01 and ∗∗∗*p* < 0.001. Two-way ANOVA followed by Tukey’s post hoc test. DAPI, 4′,6-diamidino-2-phenylindole; OSM, oncostatin M; OSMRβ, OSM receptor β subunit.

Double-immunofluorescence staining revealed that the expression of OSMRβ was observed predominantly in F4/80-positive cells (macrophages) in the skeletal muscle (Fig. 7*A*). As shown in Figure 7*B*, we confirmed the protein expression of OSMRβ in macrophages isolated from skeletal muscles by Western blot analysis. In addition, exercise-induced phosphorylation of STAT3 was observed in F4/80-positive cells (Fig. 7*C*). Intramuscular injection of OSM induced phosphorylation of STAT3 in F4/80-positive cells at 30 min after the injection (Fig. 7*D*). When isolated skeletal muscle macrophages were stimulated with OSM, phosphorylation of STAT3 was induced at 10 and 20 min after stimulation (Fig. 7, *E* and *F*).

**Figure 8:**
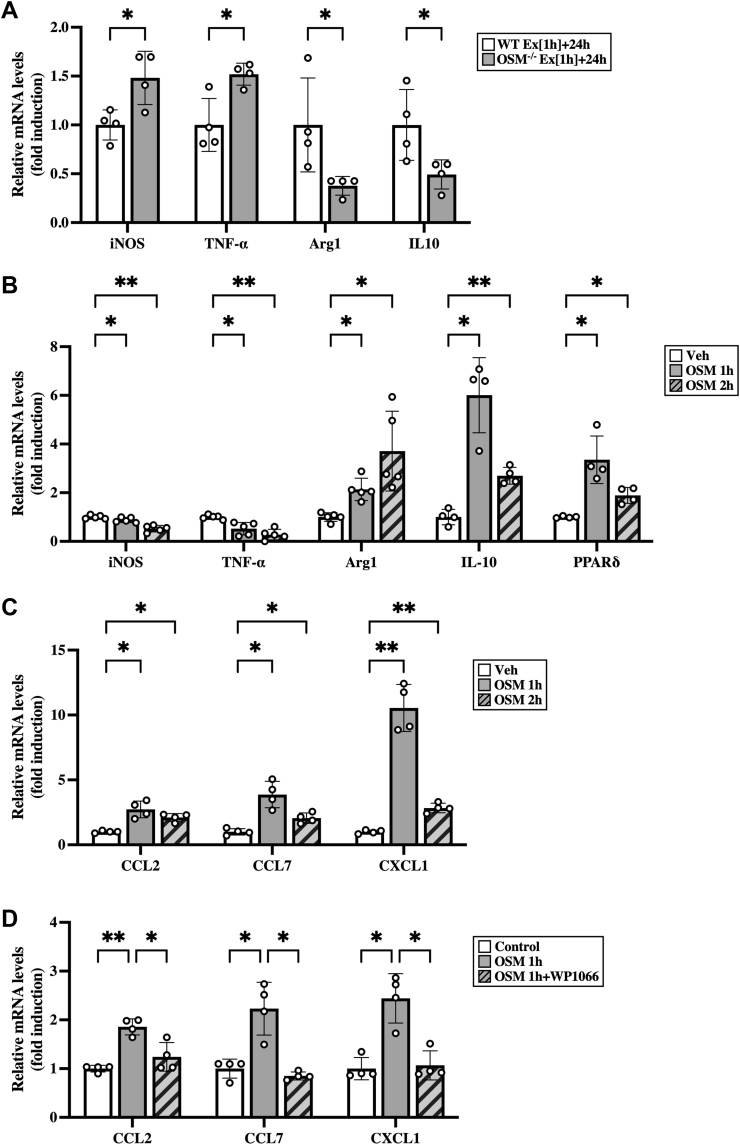
**Direct effects of OSM on skeletal muscle macrophages.***A*, expression of mRNA of M1 (iNOS and TNF-α) and M2 markers (arginase-1 [Arg1] and IL-10) in the skeletal muscle macrophages isolated from WT and OSM^−/−^ mice at 24 h after the exercise. *B*–*D*, effects of OSM on the expression of mRNA related to phenotypes of macrophage (*B*) and chemokines (*C* and *D*) in the skeletal muscle macrophages. Macrophages were isolated from skeletal muscle and then stimulated with OSM (50 ng/ml) for 1 or 2 h (*B* and *C*). Macrophages isolated from skeletal muscles were incubated with WP1066 (1 μM) for 1 h prior to OSM treatment (50 ng/ml, 1 h) (*D*). Data are expressed as mean ± SD; n = 4 to 5 per group. ∗*p* < 0.05 and ∗∗*p* < 0.01. Student’s *t* test (*A*); two-way ANOVA followed by Tukey’s post hoc test (*B*–*D*). CCL, CC chemokine ligand; CXCL1, CXC chemokine ligand 1; IL, interleukin; iNOS, inducible nitric oxide synthase; OSM, oncostatin M; TNF-α, tumor necrosis factor alpha.

To investigate the effects of OSM on the phenotypes of macrophages in skeletal muscles after the exercise, skeletal muscle macrophages were isolated from both WT and OSM^−/−^ mice at 24 h after exercise. Skeletal muscle macrophages isolated from OSM^−/−^ mice decreased the mRNA expression of M2 markers (arginase-1 and IL-10) and increased M1 markers (iNOS and TNF-α), compared with those from WT mice (Fig. 8*A*). Next, to examine the direct effects of OSM on skeletal muscle macrophages, macrophages were isolated from the skeletal muscle and stimulated with OSM. In the skeletal muscle macrophages, OSM induced the expression of markers of M2 macrophage, arginase-1, IL-10, and peroxisome proliferator–activated receptor δ (PPARδ) (Fig. 8*B*). In contrast, stimulation with OSM reduced the expression of M1 markers (iNOS and TNF-α) in the skeletal muscle macrophages (Fig. 8*B*). In addition, expression of CCL2, CCL7, and CXCL1 was increased by stimulation with OSM in skeletal muscle macrophages (Fig. 8*C*), which was completely blocked by the inhibitor of STAT3, WP1066 (Fig. 8*D*). Among these chemokines, only CXCL1 was increased by OSM in macrophages isolated from the adipose tissue (Fig. S5*C*). In hepatic macrophages, OSM increased the expression of CCL2 and CXCL1, whereas CCL7 was not detected (Fig. S5*D*).

## Discussion

Skeletal muscle produces a variety of biological active peptides and proteins, so-called myokines, in response to aerobic exercise (27). Although contraction of muscle fibers is one of the initial events in aerobic exercise, only a few myokines, including IL-6 and brain-derived neurotrophic factor, are known to be expressed from muscle fibers after aerobic exercise (28, 29). OSM, a member of the IL-6 family of cytokines, was reported to be induced in the skeletal muscle of mice during a single bout of swimming, a type of aerobic exercise (18). However, localization of exercise-induced OSM protein in the skeletal muscle remains unclear. In the present study, expression of OSM protein was increased in the skeletal muscle immediately after aerobic exercise. In addition, immunohistochemistry revealed that OSM protein was expressed in the muscle fibers of the skeletal muscle after the aerobic exercise. OSM is therefore suggested to be a novel myokine produced in muscle fibers, and it seems to play some important roles in biological events as a result of aerobic exercise.

To adapt to physiological demands in response to exercise, some alterations are induced in skeletal muscle, including metabolic changes of muscle fibers and infiltration of immune cells (30, 31). Notably, in response to resistance exercise, various immune cells, such as neutrophils, macrophages, and T-cells, infiltrate into skeletal muscle as part of the muscle repair process (32, 33, 34, 35). Crosstalk between muscle fibers and immune cells is therefore an important event in skeletal muscle adaptation to resistance exercise. Less is known about such a crosstalk between muscle fibers and immune cells during and after a single bout of aerobic exercise, although the accumulation of M2 macrophages is reported in the skeletal muscle (26). In the present study, aerobic exercise increased the number of neutrophils as well as M2 macrophages but did not significantly change the number of M1 macrophages, T-cells, or B-cells, in the skeletal muscle. The infiltration pattern of immune cells to skeletal muscle after aerobic exercise is different from that in resistance exercise, suggesting that there is unique crosstalk between muscle fibers and immune cells in response to aerobic exercise.

In numerous aspects of immune responses, chemokines play important roles in the recruitment of immune cells into tissues (36). In response to exercise, some chemokines related to the recruitment of macrophages (CCL2 and CX3CL1) and neutrophils (CXCL1) are known to be increased in skeletal muscles (37, 38, 39, 40). Immunohistochemistry in skeletal muscles reveals that some chemokines (CCL2 and CX3CL1) are produced from cells in the interstitial space between muscle fibers, including macrophages, satellite cells, and endothelial cells, after exercise (37, 38). On the other hand, CCL2 and CXCL1 have been shown to be produced from contracted C2C12 myotubes (41, 42, 43, 44), suggesting that muscle fibers may produce chemokines through their contraction during exercise. The regulatory mechanisms underlying the expression of chemokines after exercise still therefore require deeper elucidation. In the present study, the chemokines related to the recruitment of macrophages (CCL2, CCL7, and CX3CL1) and neutrophils (CXCL1) were increased in the skeletal muscle after the aerobic exercise, whereas the increased expression of these chemokines was attenuated in OSM^−/−^ mice. Among these chemokines, CCL2, CCL7, and CXCL1 were induced by the intramuscular injection of OSM in the skeletal muscle (*in vivo*) and in OSM-stimulated macrophages taken from the skeletal muscle through the activation of STAT3 (*in vitro*). These results suggest that aerobic exercise induced such chemokines in the skeletal muscle macrophages at least in part through OSM.

Macrophages are highly plastic cells that are mainly divided into two phenotypes, M1 and M2 (45, 46). In the present study, M2 macrophages, but not M1 macrophages, were increased by aerobic exercise in the skeletal muscle. In addition, M2 macrophages were decreased and M1 macrophages were increased in the skeletal muscle of OSM^−/−^ mice after aerobic exercise, compared with those of WT mice. These results suggest that OSM is important to polarize the phenotypes of macrophages to M2 type after the aerobic exercise. Furthermore, OSM directly induced the expression of PPARδ as well as arginase-1, a marker of M2 macrophages in skeletal muscle macrophages. The polarization of M2 type is reported to regulate by PPARδ in bone marrow–derived macrophages (47), and OSM may directly polarize the phenotypes of macrophages to M2 type through the induction of PPARδ in the skeletal muscle after aerobic exercise. On the other hand, OSM induced the expression of IL-10 in the skeletal muscle macrophages, and IL-10 is well known to change the phenotypes of macrophages from M1 to M2 type (48, 49, 50). Such direct and indirect effects of OSM may synergistically contribute to the phenotypic determination of macrophages after aerobic exercise.

The roles of M2 macrophages and neutrophils that accumulate after aerobic exercise are unclear. However, the depletion of M2 macrophages in the skeletal muscle by using clodronate liposome attenuates aerobic exercise–induced increase in insulin sensitivity (26). In addition, walking exercise–induced neutrophil accumulation is associated with glycemic control. Neutrophil-induced IL-1β stimulates the translocation of glucose transporter 4 in the muscle fibers, which leads to the increase in insulin-stimulated glucose transport (51). Macrophages and neutrophils induced by the aerobic exercise are suggested to be associated with the increase in insulin sensitivity in the skeletal muscle. Further studies will be required to better understand the functional roles of these immune cells in the skeletal muscle after aerobic exercise.

In conclusion, M2 macrophages and neutrophils were shown to be accumulated in skeletal muscles after aerobic exercise. OSM directly regulates the recruitment of these immune cells through the expression of some chemokines (CCL2, CCL7, and CXCL1). It is strongly suggested that OSM is a key myokine that regulates the accumulation of immune cell in the skeletal muscle after aerobic exercise.

## Experimental procedures

### Animals

Eight-week-old male C57BL/6J mice were purchased from Nihon SLC. WT and OSM^−/−^ littermates were obtained from our breeding colony using heterozygous breeding pairs. Estrogen is reportedly one of the factors that affect accumulation of immune cells in the skeletal muscle (52, 53). To avoid the confounding effects of the hormonal fluctuation because of the female estrous cycle, only male mice were used in the present study. All mice were housed in specific pathogen-free facilities under light (12-h light/dark)-, temperature (22–25 °C)-, and humidity (50–60% relative humidity)-controlled conditions. Mice were allowed free access to food and water. All experimental procedures were approved by the Wakayama Medical University Animal Research Committee and carried out in accordance with the National Institutes of Health (NIH) Guide for the Care and Use of Laboratory Animals (NIH publication no.: 80-23, revised 1978) and the Wakayama Medical University in-house guidelines for the care and use of laboratory animals.

### Protocol of aerobic exercise

Mice were randomly assigned to either the sedentary or the aerobic exercise groups. A motor-driven treadmill (modular enclosed metabolic treadmill for mice; Columbus Instruments) and an open circuit calorimeter (Oxymax System, Columbus Instruments) were used in exercise experiments. The treadmill was equipped with a small electric shock–delivering grid that was used to encourage running during the exercise protocol. Electric shock intensity was set to 0.1 mA. VO_2_max in 8-week-old male C57BL/6J mice was measured as described elsewhere (54, 55). All mice were acclimated to the treadmill for 2 days prior to the exercise bout (day 1: 5 min rest on the treadmill; day 2: 5 min rest on the treadmill followed by 5 min at the speed of 5 m/min). On day 3, mice in the aerobic exercise group were acclimated to treadmill running at a speed of 5 m/min for 5 min followed by 10 m/min for 5 min with 0° inclination. Mice were then forced to run on a treadmill at a speed of 15 m/min with 0° inclination for 0.5 or 1 h (%VO_2_max: 73.7 ± 1.8% in C57BL/6J mice). Mice in the sedentary groups remained in their cages in the treadmill room throughout the bouts of exercise without food or water. Mice were started to run on the treadmill at 15:00.

### Intramuscular injection with OSM

Injection of OSM in the skeletal muscle was performed as described elsewhere (56). To investigate the direct effects of OSM on skeletal muscle, C57BL/6J mice were injected with recombinant mouse OSM (R&D Systems) into both sides of calf muscle (6.25 ng/g body weight in each side). This dose of OSM was based on our previous study (15). C57BL/6J mice injected with vehicle in both sides of calf muscle were used as control mice. One, 2, or 24 h after the injection, total RNAs and proteins were extracted from the calf muscles for quantitative real-time PCR and Western blot analysis, respectively. About 30 min or 24 h after the injection, the calf muscles were taken for immunohistochemistry. In this experiment, mice were not exercised before the injection of OSM so that the direct effects of OSM could be investigated *in vivo* because such exercise would induce the expression of OSM in the skeletal muscle.

### Measurement of blood glucose

Blood glucose was measured with some modifications as described previously (15). Mice were fasted for 4 h (14:00–18:00 h) to remove the effects of food intake on glucose metabolism, and blood was taken from the retro-orbital plexus. Blood glucose levels were measured by a glucose measurement device (Glucocard GT-1640; Arkray).

### Insulin signaling analysis

Insulin signaling analysis was performed as previously described (17). To evaluate insulin signaling, mice fasted for 4 h (14:00–18:00 h) were intraperitoneally injected with human insulin (1 milliunits/g of body weight) at 18:00 h. About 10 min later, calf muscles were taken for Western blot analysis. Tissue lysates were prepared as described.

### Tissue preparation

For immunohistochemistry, mice were deeply anesthetized with isoflurane and transcardially perfused with ice-cold 0.85% NaCl followed by ice-cold Zamboni’s fixative (2% paraformaldehyde and 0.2% picnic acid in 0.1 M PBS). Both sides of the calf muscles (gastrocnemius, plantaris, and soleus) was quickly removed, postfixed in the same fixative at 4 °C for 3 h, and cryoprotected in 20% sucrose in 0.1 M PBS. All specimens were embedded with optimal cutting temperature compound, frozen rapidly in cold *n*-hexane on dry ice, and stored at −80 °C.

For Western blot analysis and quantitative real-time PCR, mice were sacrificed by cervical dislocation under deep isoflurane anesthesia. Both sides of the calf muscles were rapidly removed, immediately frozen in liquid nitrogen, and stored at −80 °C.

### Isolation of resident macrophages from skeletal muscle, adipose tissue, and liver, and stimulation with OSM

Isolation of skeletal muscle macrophages was performed as described elsewhere (57). C57BL/6J mice were deeply anesthetized with isoflurane, and both sides of the calf muscle (gastrocnemius, plantaris, and soleus), the epididymal adipose tissue, and the liver were quickly removed. The calf muscle was minced into fine pieces and digested with collagenase type B (2 mg/ml; Sigma–Aldrich) and collagenase type D (1 mg/ml; Sigma–Aldrich) dissolved in Dulbecco's modified Eagle's medium (DMEM; Invitrogen) using a gentleMACS Dissociator (Miltenyi Biotec). Collagenase type II (1 mg/ml; Sigma–Aldrich) and collagenase type IV (1 mg/ml; Sigma–Aldrich) were used to digest the adipose tissue and the liver, respectively. The samples were then passed through a nylon mesh (100 μm pore size; BD Biosciences) and centrifuged at 1200 rpm for 5 min. The cells in the pellets were resuspended in DMEM supplemented with 2% fetal calf serum and incubated with anti-CD16/CD32 antibodies (BD Biosciences) to block Fc binding. Macrophages were sorted using anti-F4/80 MicroBeads UltraPure (Miltenyi Biotec) and the autoMACS Pro Separator (Miltenyi Biotec). The sorted cells were cultured in DMEM supplemented with 10% fetal calf serum, 100 U/ml of penicillin (Invitrogen), and 100 μg/ml of streptomycin (Invitrogen) at 37 °C for 24 h in a humidified atmosphere of 5% CO_2_. These cells were then treated with vehicle or 50 ng/ml of recombinant mouse OSM (R&D Systems) for 10 min, 20 min, 1 h, or 2 h. The dose of OSM in *in vitro* study was determined according to our previous report (58). For the experiments using STAT3 inhibitor in skeletal muscle macrophages, WP1066 (Merck Millipore), the cells were incubated with WP1066 (1 μM) for 1 h prior to OSM treatment (50 ng/ml for 1 h).

### Flow cytometry

Flow cytometry was performed as described previously (58). Briefly, the cells isolated from the calf muscle (gastrocnemius, plantaris, and soleus) were incubated with anti-CD16/CD32 antibodies (1:100 dilution; BD Biosciences) to block Fc binding at 4 °C for 5 min, followed by incubation with fluorescently labeled primary antibodies or isotype-matched control antibodies at 4 °C for 30 min. The FITC-conjugated anti-CD45 antibody (clone ID: 30-F11), phycoerythrin (PE)-conjugated anti-CD11b antibody (clone ID: M1/70), allophycocyanin-conjugated anti-F4/80 antibody (clone ID: BM8), Super Bright 600-conjugated anti-Ly6G antibody(clone ID: 1A8-Ly6g), PE-Cy7-conjugated anti-CD206 antibody (clone ID: MR6F3), allophycocyanin-eFluor780-conjugated anti-CD11c antibody (clone ID: N418), FITC-conjugated anti-CD3e antibody (clone ID: 145-2C11), and eFluor506-conjugated anti-B220 antibody (RA3-6B2) were purchased from eBiosciences. The stained cells were analyzed using a CytoFLEX (Beckman Coulter). Dead cells were removed from the analysis using 7-aminoactinomycin D staining (eBioscience). The results of flow cytometry were analyzed with FlowJo software (Tree Star). The plot of a forward scatter *versus* side scatter was used as the first gate to gate out aggregates and debris (Fig. S6). To identify individual live cells, the events were then gated based on side scatter *versus* 7-aminoactinomycin D (Fig. S6). Next, the CD45, F4/80, and CD11b-triple-positive cells were selected as macrophages in the skeletal muscle (Fig. S6). M1 and M2 macrophages were identified as CD11c- and CD206-positive cells, respectively, in the macrophage fraction as previously described (57). Single color controls were used to set the compensation and gates.

### Immunohistochemistry

Immunofluorescence staining of the skeletal muscle was performed as described previously with some modifications (15). Six-micrometer-thick frozen sections were cut on a cryostat. For indirect immunofluorescence staining, sections were incubated with 5% normal donkey serum (Jackson ImmunoResearch Laboratories) at room temperature (RT) for 1 h. The sections were then incubated with primary antibodies at 4 °C overnight. Primary antibodies were used at the following dilutions: goat anti-OSM antibody (diluted at 1:100; catalog no.: ab10843, R&D Systems); goat anti-OSMRβ antibody (diluted at 1:100; catalog no.: AF662, R&D Systems); rat anti-F4/80 antibody (diluted at 1:100; catalog no.: ab6640, Abcam); rat anti-CD206 antibody (diluted at 1:400; clone ID: MR5D3, Serotec); rat anti-CD11c antibody (diluted at 1:100; catalog no.: ab52632, Abcam); rabbit anti-MPO antibody (diluted at 1:100; catalog no.: ab9535, Abcam); rat anti-laminin α-2 antibody (diluted at 1:100; clone ID: 4H8-2, Santa Cruz Biotechnology); rabbit anti-laminin antibody (diluted at 1:100; catalog no.: L9393, Sigma–Aldrich); rabbit anti–phospho-STAT3 antibody (diluted at 1:100; catalog no.: 9131, Cell Signaling Technology), rabbit antislow twitch MyHC (type I) antibody (diluted at 1:100; catalog no.: ab234431, Abcam), mouse antifast twitch MyHC (type IIa) antibody (diluted at 1:100; clone ID: SC-71, Merck Millipore), rat antifast twitch MyHC (type IIb) antibody (diluted at 1:100; clone ID: 2G72F10, Merck Millipore), rabbit antifast twitch MyHC (type IIx) antibody (diluted at 1:100; catalog no.: ab91506, Abcam). After being washed, the sections were incubated with Cy2- or Cy3-conjugated secondary antibodies (Jackson ImmunoResearch) at RT for 1 h. For direct immunofluorescence staining, sections were incubated with anti-CD16/32 antibody (diluted at 1:100; BD Biosciences) to block Fc binding at 4 °C for 1 h, followed by incubation with fluorescently labeled primary antibodies at 4 °C overnight. Primary antibodies were used at the following dilutions: FITC-conjugated anti-CD3e antibody (diluted at 1:100; clone ID: 145-2C11, eBioscience) and PE-conjugated anti-B220 antibody (diluted at 1:100; clone ID: RA3-6B2, BD Biosciences). All sections were counterstained with 4′,6-diamino-2-phenylindole. To reduce autofluorescence, sections were incubated with TrueVIEW Autofluorescence Quenching Kit (Vector Laboratories) according to the manufacturer's instructions. Immunofluorescence images were acquired using an epifluorescence microscope (Olympus) equipped with a digital charge-coupled device camera (Olympus).

The number of F4/80-, CD11c-, CD206-, MPO-, CD3e-, and B220-positive cells was quantified in relation to the total area of the cross section of the calf muscle. After immunohistochemical staining to laminin, whole images of the muscle sections were acquired on a fluorescence microscope (BZ-X810; Keyence) using image stitching software (BZ-H4XD and BZ-H4A; Keyence). The total area in each muscle section was measured using ImageJ (US NIH). The number of positive cells was normalized by the total area of the muscle section. Six coronal sections of the calf muscle containing soleus, gastrocnemius, and plantaris muscles at 120 μm intervals were used for quantification.

### Western blot analysis

Western blot analysis was performed with some modifications, as previously described (15). Lysates from the calf muscles were prepared using a raioimmunoprecipitation assay buffer (Upstate Biotechnology) containing a protease inhibitor cocktail (Upstate Biotechnology), 1 mM sodium orthovanadate, 1 mM sodium fluoride, and 1 mM phenylmethylsulfonyl fluoride. The protein concentrations of the lysates were determined using the Bicinchoninic Acid Protein Assay kit (Pierce). About 20 μg of protein samples were separated by SDS-PAGE and transferred to Immun-Blot low fluorescence polyvinylidene difluoride membranes (Bio-Rad). After blocking with EveryBlot Blocking Buffer (Bio-Rad) at RT for 5 min, the blotted membranes were incubated with primary antibodies at 4 °C overnight. Primary antibodies were used at the following dilutions: rabbit anti-iNOS antibody (diluted at 1:2000; catalog no.: ab15323, Abcam); rabbit anti-TNF-α antibody (diluted at 1:2000; catalog no.: ab6671, Abcam); sheep anti-arginase-1 antibody (diluted at 1:2000; catalog no.: AF5868, R&D Systems); goat anti-IL-10 antibody (diluted at 1:1000; catalog no.: AF519, R&D Systems); goat anti-OSM antibody (diluted at 1:1000, R&D Systems); goat anti-CCL2 antibody (diluted at 1:1000, catalog no.: AF-479-NA, R&D Systems); goat anti-CCL7 antibody (diluted at 1:1000, catalog no.: AF-456-NA, R&D Systems); goat anti-CXCL1 antibody (diluted at 1:1000, catalog no.: AF-453-NA, R&D Systems); goat anti-OSMRβ antibody (diluted at 1:1000, R&D Systems); rabbit anti–phospho-STAT3 antibody (diluted at 1:1000); and rabbit anti–phospho-Akt antibody (diluted 1:2000, catalog no.: 4060, Cell Signaling Technology). Thereafter, the membranes were incubated with IRDye 800CW-conjugated donkey antigoat immunoglobulin G (IgG) antibody (diluted at 1:20,000, LI-COR Biosciences), StarBright Blue 700-conjugated goat anti-rabbit IgG antibody (diluted at 1:5000, Bio-Rad), or horseradish peroxidase–conjugated donkey antisheep IgG antibody (diluted at 1:10,000, Jackson ImmunoResearch). For the experiments using horseradish peroxidase–conjugated secondary antibody, labeled proteins were detected with chemiluminescence using the enhanced chemiluminescence detection reagent (GE Healthcare). Images were obtained using a ChemiDoc Touch Imaging System (Bio-Rad) according to the manufacturer's instructions. Subsequently, the blotted membranes were stripped in 0.25 M glycine, pH 2.5, at RT for 10 min and incubated with hFAB Rhodamine–conjugated anti-β-tubulin antibody (diluted at 1:10,000, Bio-Rad), rabbit anti-STAT3 antibody (diluted at 1:1000, catalog no.: sc-482, Santa Cruz Biotechnology), or rabbit anti-Akt antibody (diluted 1:2000, catalog no.: 9272, Cell Signaling Technology) at RT for 1 h.

### Quantitative real-time PCR

Quantitative real-time PCR was performed as described previously with some modifications (15). Briefly, total RNAs from the calf muscle, epididymal adipose tissue, and liver, and from cultured macrophages were prepared using TRI reagent (Molecular Research Center). Using the total RNA, the complementary DNA was synthesized with High Capacity complementary DNA Reverse Transcription Kit (Applied Biosystems). Quantitative real-time PCR for each gene was performed using Rotor Gene Q (Qiagen) and Rotor Gene Probe PCR Kits (Qiagen). The following TaqMan Gene Expression Assays (Applied Biosystems) were used: OSM (Mm01193966_m1), iNOS (Mm00440502_m1), arginase-1 (Mm00475988_m1), TNF-α (Mm00443258_m1), IL-10 (Mm00439616_m1), CCL2 (Mm00441242_m1), CCL7 (Mm00443113_m1), CX3CL1 (Mm00436454_m1), CXCL1 (Mm04207460_m1), PPARδ (Mm00803184_m1), and 18S (Hs99999901_s1). The PCR amplification protocol was as follows: initial denaturation at 95 °C for 10 min and then 40 cycles of 95 °C for 10 s and 60 °C for 45 s. The expression of each gene was normalized by 18S ribosomal RNA expression and analyzed using ΔΔCT method.

### Statistical analysis

Results are shown as mean ± SD. Comparison between the two groups was analyzed by Student’s *t* test. For multiple-group comparisons, ANOVA was used followed by Tukey’s post hoc test. Data were analyzed with GraphPad Prism 9 (GraphPad Software, Inc). The criterion for statistical significance was *p* < 0.05.

## Data availability

All data generated or analyzed during this study are included in this article or are available from the corresponding author (Yoshihiro Morikawa, Wakayama Medical University, E-mail: yoshim@wakayama-med.ac.jp) upon reasonable request.

## Supporting information

This article contains supporting information (18, 59) .

## Conflict of interest

The authors declare that they have no conflicts of interest with the contents of this article.
