# Supplementary material for: Essential roles of the cytokine oncostatin M in crosstalk between muscle fibers and immune cells in skeletal muscle after aerobic exercise
Source: J Biol Chem. 2022 Nov 9;298(12):102686. doi: 10.1016/j.jbc.2022.102686 (PMC9720348; doi:10.1016/j.jbc.2022.102686)
Supplement: Supporting information [file mmc1.docx]

**Supporting information**

**Essential roles of the cytokine oncostatin M in crosstalk between muscle fibers and immune cells in skeletal muscle after aerobic exercise**

Tadasuke Komori, Yoshihiro Morikawa

**Supporting Experimental procedures**

*Swimming exercise*

This procedure was performed as described elsewhere (18). Mice were randomly assigned to either the sedentary or the exercise groups. Mice swam in 37°C warm water for 60 min and were euthanized immediately after the swimming bout. In the sedentary groups, mice remained in their cages in the same room with exercised mice throughout the bouts of exercise without food or water. Mice were started to swim at 15:00.

*Treadmill running exercise with gentle encouragement*

This procedure was performed as described elsewhere (59). Mice were randomly assigned to either sedentary or exercise groups. A motor-driven treadmill (KN-73, Natsume, Tokyo, Japan) was used in exercise experiments. All mice were acclimated to the treadmill for 2 days prior to the exercise bout (Day 1: 5 min rest on the treadmill; Day 2: 5 min rest on the treadmill followed by 5 min at the speed of 5 m/min). On Day 3, mice in the aerobic exercise group were acclimated to treadmill running at a speed of 5 m/min for 5 min, followed by 10 m/min for 5 min with 0° inclination. Then the mice were forced to run on a treadmill at a speed of 15 m/min with 0° inclination for 1 h. Use of the shock grid was replaced by encouragement by a human operator using gentle tapping or touching with a tongue depressor as needed (59). Mice in the sedentary groups remained in their cages in the treadmill room throughout the bouts of exercise without food or water. Mice were started to run on the treadmill at 15:00.

**Supporting Figures and Figures Legends**

**
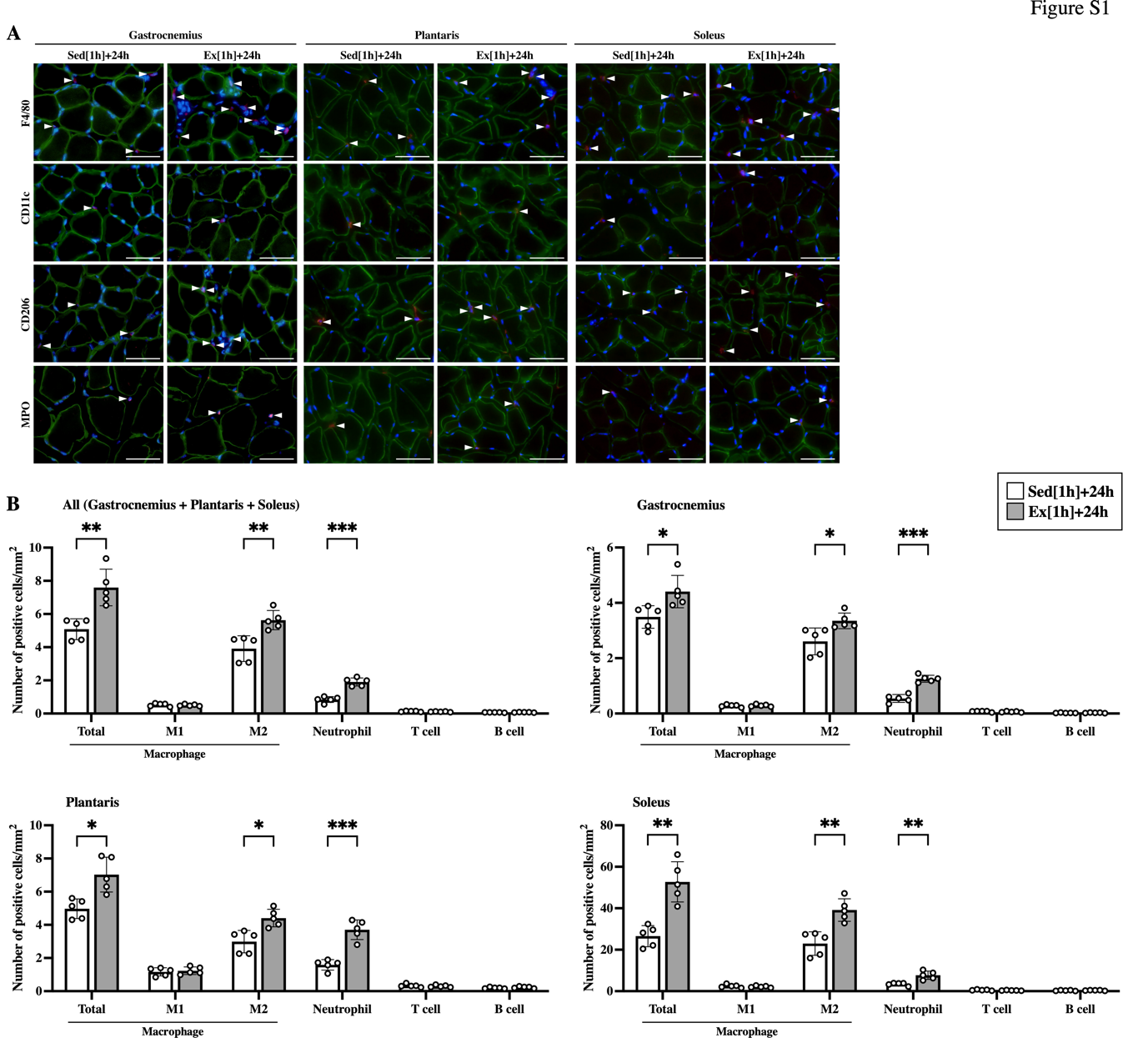
**

**Figure S1. Distribution of exercise-induced immune cells in the skeletal muscle.** (A) Representative images of total macrophages (F4/80-positive cells, red; arrow heads), M1 macrophages (CD11c-positive cells, red; arrow heads), M2 macrophages (CD206-positive cells, red; arrow heads), and neutrophils (MPO-positive cells, red; arrow heads) in the skeletal muscle of sedentary mice (Sed[1h]+24h) and mice at 24 h after the exercise (Ex[1h]+24h). The sections were co-stained with an antibody against laminin to identify the muscle fibers (green). The sections were counterstained with DAPI (blue). Scale bars = 50 μm. (B) Quantitative analysis of the number of total macrophages, M1 macrophages, M2 macrophages, neutrophils, T cells, and B cells in the skeletal muscle at 24 h after the exercise. Data are expressed as mean ± SD; n = 5 per group. **p* < 0.05, ***p* < 0.01, ****p* < 0.001. Student’s *t* test.

**
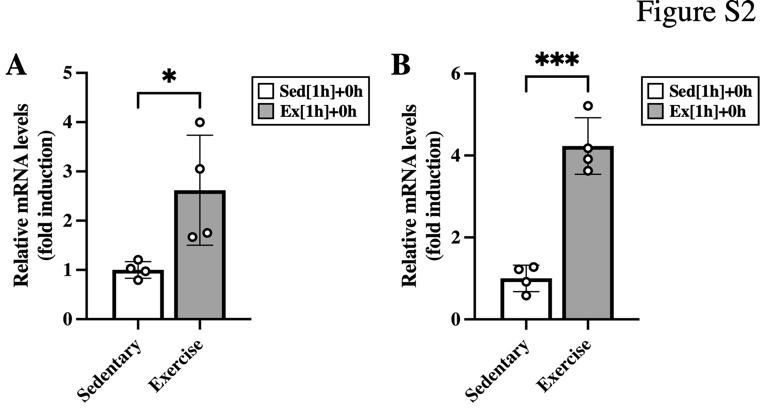
**

**Figure S2. The effects of swimming exercise and treadmill running exercise without electrical stimulation on the expression of OSM in the skeletal muscle.** C57BL/6J mice were subjected to a single bout of swimming exercise (A) or treadmill running exercise at a speed of 15 m/min for 1 h (B). Gene expression of OSM in the skeletal muscle of mice immediately (Ex[1h]+0h) after the exercise. Data are expressed as mean ± SD; n = 4 per group. **p* < 0.05, ****p* < 0.001. Student’s *t* test.

**
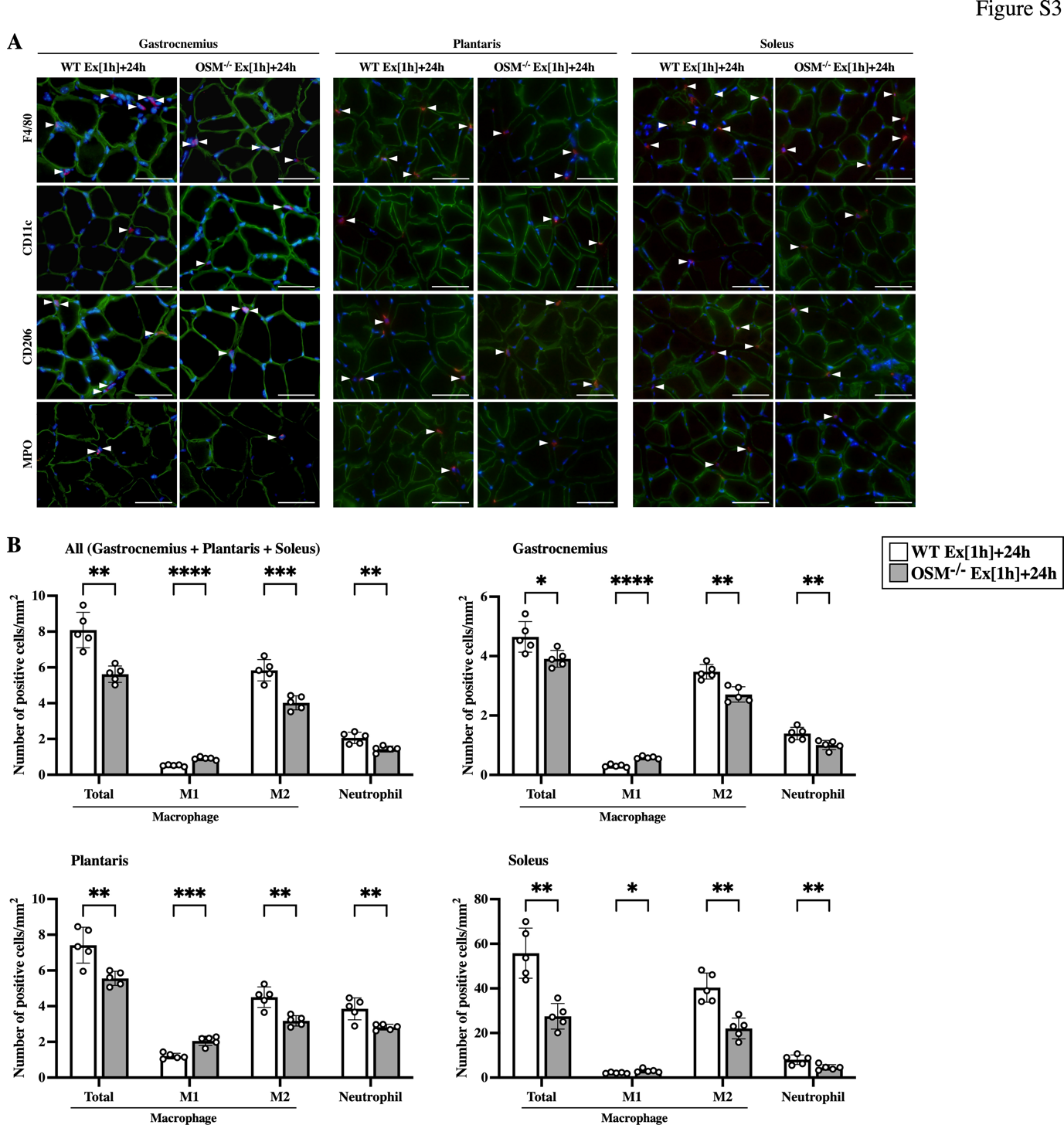
**

**Figure S3.** **Distribution of exercise-induced accumulation of macrophages and neutrophils in the skeletal muscle of OSM^-/-^ mice.** (A) Representative images of total macrophages (F4/80-positive cells, red; arrow heads), M1 macrophages (CD11c-positive cells, red; arrow heads), M2 macrophages (CD206-positive cells, red; arrow heads), and neutrophils (MPO-positive cells, red; arrow heads) in the skeletal muscle of WT (WT Ex[1h]+24h) and OSM^-/-^ mice (OSM^-/-^ Ex[1h]+24h) at 24 h after the exercise. Sections were co-stained with an antibody against laminin to identify the muscle fibers (green). The sections were counterstained with DAPI (blue). Scale bars = 50 μm. (B) Quantitative analysis of the number of total macrophages, M1 macrophages, M2 macrophages, and neutrophils in the skeletal muscle of WT and OSM^-/-^ mice at 24 h after the exercise. Data are expressed as mean ± SD; n = 5 per group. **p* < 0.05, ***p* < 0.01, ****p* < 0.001, *****p* < 0.0001. Student’s *t* test.

**
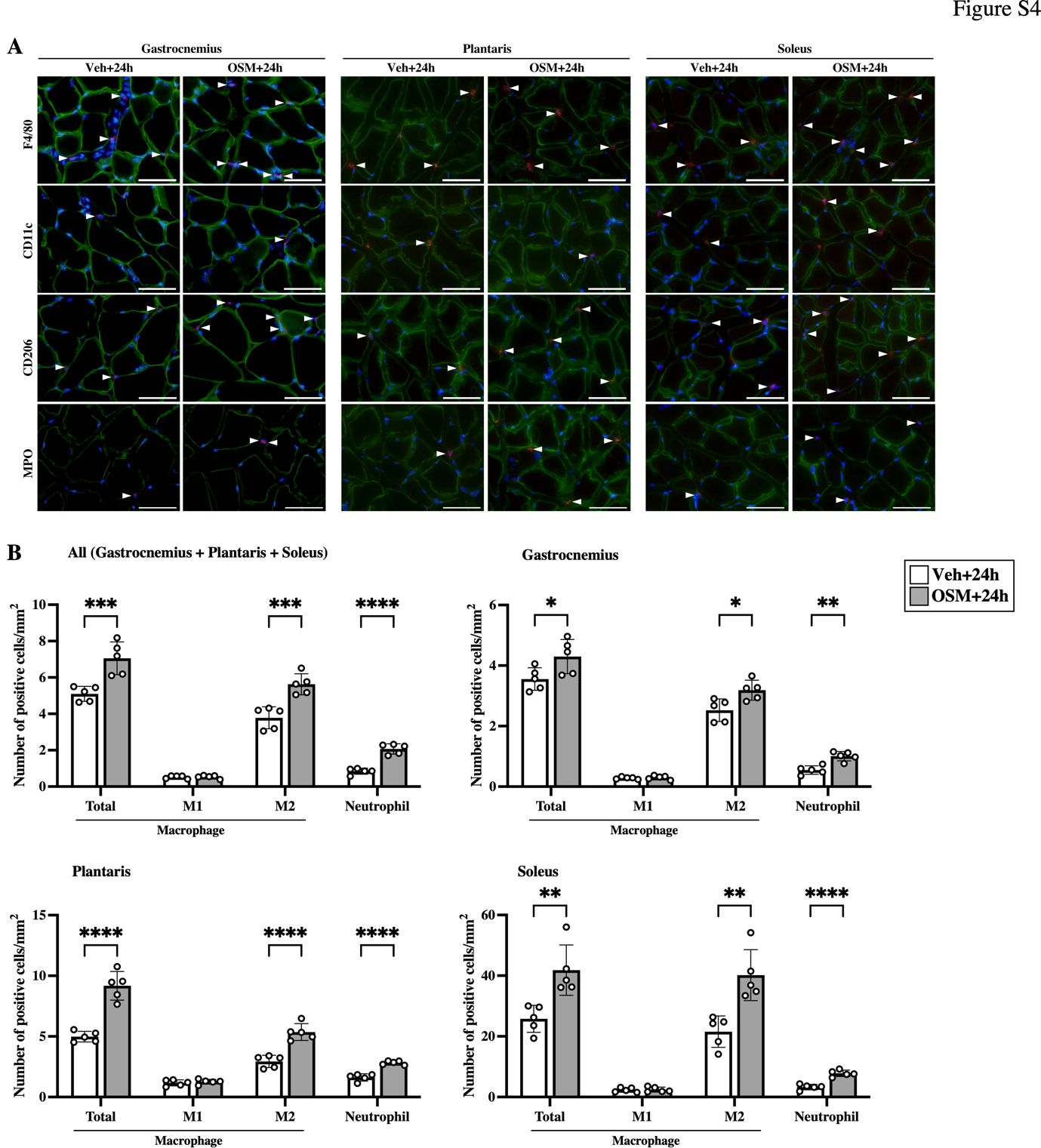
**

**Figure S4.** **Distribution of macrophages and neutrophils accumulated by OSM in the skeletal muscle.** (A) Representative images of total macrophages (F4/80-positive cells, red; arrow heads), M1 macrophages (CD11c-positive cells, red; arrow heads), M2 macrophages (CD206-positive cells, red; arrow heads), and neutrophils (MPO-positive cells, red; arrow heads) in the skeletal muscle of mice at 24 h after the injection of vehicle (Veh+24h) or OSM (OSM+24h). Sections were co-stained with an antibody against laminin to identify the muscle fibers (green). The sections were counterstained with DAPI (blue). Scale bars = 50 μm. (B) Quantitative analysis of the number of total macrophages, M1 macrophages, M2 macrophages, neutrophils in the skeletal muscle at 24 h after the injection of OSM. Data are expressed as mean ± SD; n = 5 per group. **p* < 0.05, ***p* < 0.01, ****p* < 0.001, *****p* < 0.0001. Student’s *t* test.

**
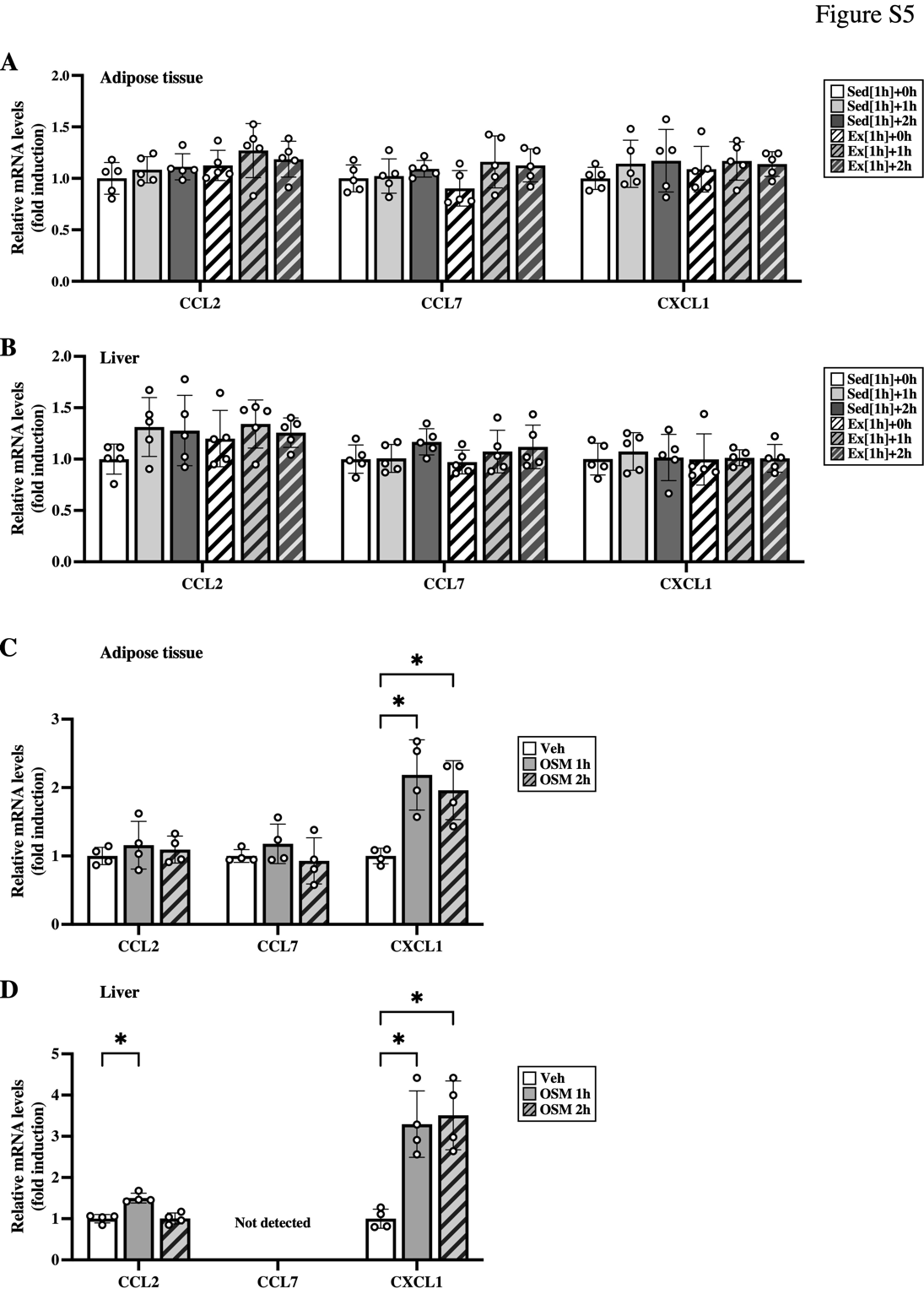
**

**Figure S5. Extramuscular effects of exercise on chemokine expression.**  (A and B) Expression of chemokines in the adipose tissue (A) and liver (B) after the exercise. C57BL/6J mice were subjected to a single bout of treadmill running exercise at a speed of 15 m/min for 1 h. (C and D) Effects of OSM on the expression of chemokines in macrophages isolated from the adipose tissue (C) and liver (D). Isolated macrophages were stimulated with OSM (50 ng/ml) for 1 or 2 h. Data are expressed as mean ± SD; n = 5 per group (A and B); n = 4 per group (C and D). **p* < 0.05. Two-way ANOVA followed by Tukey’s post hoc test.


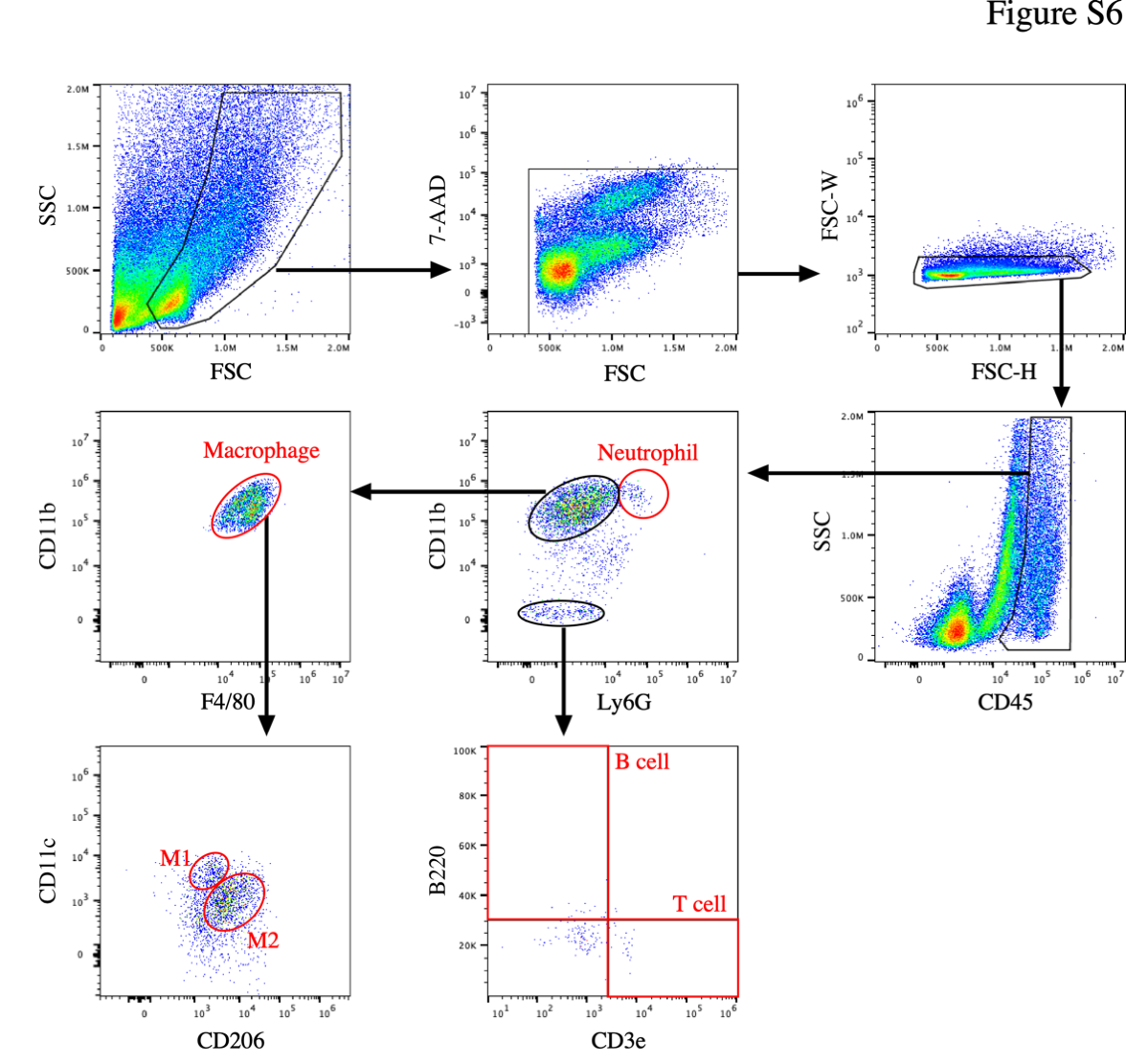


**Figure S6. Gating strategy for flow cytometry.** Identification of immune cells in the skeletal muscle by flow cytometry. Cells were isolated with collagenase treatment of the skeletal muscle and labeled with antibodies against CD45, CD11b, Ly6G, F4/80, CD11c, CD206, CD3e, and B220. Positive staining was identified using isotype control- and single-stained samples. First, cell debris was excluded from the analysis using a polygon gate (black) in the FSC/SSC plot (upper left panel). Second, a rectangle gate (black) in the FSC/7-AAD plot was used to eliminate dead cells (upper middle panel). Third, FSC-H/FSC-W plot was used to eliminate doublet cells (upper right panel). After CD45-positive cells were gated using a polygon gate (black) in the CD45/SSC plot to select the leukocyte population (middle right panel), neutrophils were selected in the Ly6G/CD11b plot (red, middle panel). Total macrophages were gated using an ellipsoid gate (red) in the F4/80/CD11b plot (middle left panel). Then, M1 and M2 macrophages were gated using ellipsoid gate (red) in the CD206/CD11c plot (lower left panel). T-cells and B-cells were gated using four-quadrant gates (red) in the CD3e/B220 plot (lower middle panel).
